# The protein methyltransferase *Tr*SAM inhibits cellulase gene expression by interacting with the negative regulator ACE1 in *Trichoderma reesei*

**DOI:** 10.1038/s42003-024-06072-1

**Published:** 2024-03-28

**Authors:** Zhihua Zhu, Gen Zou, Shunxing Chai, Meili Xiao, Yinmei Wang, Pingping Wang, Zhihua Zhou

**Affiliations:** 1 CAS-Key Laboratory of Synthetic Biology, CAS Center for Excellence in Molecular Plant Sciences, Institute of Plant Physiology and Ecology, Chinese Academy of Sciences, 300 FengLin Rd, Shanghai, 200032 China; 2 University of Chinese Academy of Sciences, Beijing, 100049 China; 3 Shanghai Key Laboratory of Agricultural Genetics and Breeding, Institute of Edible Fungi, Shanghai Academy of Agricultural Science, 1000 Jinqi Rd, Shanghai, 201403 China

**Keywords:** Fungi, Post-translational modifications

## Abstract

Protein methylation is a commonly posttranslational modification of transcriptional regulators to fine-tune protein function, however, whether this regulation strategy participates in the regulation of lignocellulase synthesis and secretion in *Trichoderma reesei* remains unexplored. Here, a putative protein methyltransferase (*Tr*SAM) is screened from a *T. reesei* mutant with the ability to express heterologous β-glucosidase efficiently even under glucose repression. The deletion of its encoding gene *trsam* causes a significant increase of cellulase activities in all tested *T. reesei* strains, including transformants of expressing heterologous genes using *cbh1* promotor. Further investigation confirms that *Tr*SAM interacts with the cellulase negative regulator ACE1 via its amino acid residue Arg^383^, which causes a decrease in the ACE1-DNA binding affinity. The enzyme activity of a *T. reesei* strain harboring ACE1^R383Q^ increases by 85.8%, whereas that of the strains with *trsam* or *ace1* deletion increases by more than 100%. By contrast, the strain with ACE1^R383K^ shows no difference to the parent strain. Taken together, our results demonstrate that *Tr*SAM plays an important role in regulating the expression of cellulase and heterologous proteins initiated by *cbh1* promotor through interacting with ACE1^R383^. Elimination and mutation of *Tr*SAM and its downstream ACE1 alleviate the carbon catabolite repression (CCR) in expressing cellulase and heterologous protein in varying degrees. This provides a new solution for the exquisite modification of *T. reesei* chassis.

## Abstract (web summary)

The methyltransferase *Tr*SAM from *Trichoderma reesei* is suggested to have an important role in regulating the expression of cellulase and heterologous proteins initiated by *cbh1* promotor through interacting with ACE1^R383^.

## Introduction

The filamentous fungus *Trichoderma reesei* (teleomorph *Hypocrea jecorina*) is one of the most well-known industrial strains used for producing cellulase and hemicellulase, which release fermentable soluble sugars from plant biomass^1,2^. The high production levels of lignocellulose-degrading enzymes in *T. reesei* are dependent on induction by cellulose- and hemicellulose-containing plant polysaccharide mixtures. Besides, *T. reesei* is also an ideal chassis for the expression of various heterologous proteins^3^. The highly efficient expression level of target proteins may play a key role in reducing the cost of bioprocessing. Deleting and/or overexpressing transcription factors (TFs) is a straightforward method to increase the target protein yield of *T. reesei*.

One of the most extensively studied TFs is the negative regulator Cre1^4,5^, an ortholog of Mig1 from *Saccharomyces cerevisiae*^6^, a regulator mediating carbon catabolite repression (CCR) in filamentous fungi. Glucose-induced CCR is a mechanism to maintain metabolic efficiency of an organism by ensuring the utilization of carbon resource with the highest catabolic performance^7^. It indicated that CCR is conserved in fungi, although its regulatory mechanism is not fully understood^8^. In industrial filamentous fungi, CCR is also triggered by the Mig1/2 homologs, Cre1/CreA^9^. The availability of glucose supplied from the culture medium leads to the transcriptional down-regulation of genes responsible for the utilization of alternative carbon sources, such as cellulose, hemicellulose and other stubborn carbon sources. It was reported that D-glucose-6-phosphate (the intercellular metabolite of extracellular D-glucose) was associated with CCR in *T. reesei*, however, its specific regulatory mechanism was still barely known with some controversial evidences^10–12^.

The global activator XYR1 is obligatory for most cellulase and hemicellulase gene expression^13,14^. Other recognized TFs are the positively acting ACE2^5,15^ and HAP2/3/5 complex^16^, and the negatively acting ACE1^17^. In addition, BglR^18^ is identified as a new TF that upregulates the expression of specific genes encoding β-glucosidases in *T. reesei*, and the putative methyltransferase Lae1^19^ is thought to be essential for cellulase and hemicellulase production in *T. reesei*, although the mechanism remains unclear. ACE3 has been recently identified as indispensable for cellulase and xylanase activity in *T. reesei*^20^. Based on these TFs and their consensus sequences, artificially optimized promoters are also widely used to overexpress endogenous and heterologous proteins in *T. reesei*. However, the functions of most TFs in the *T. reesei* genome are still unknown. In addition, the TFs themselves are subject to multiple modifications and regulatory mechanisms^5,21,22^. Incomplete knowledge of the transcriptional regulatory networks for the synthesis and secretion of hydrolytic enzymes still hampers the systematic improvement of endogenous and heterologous protein production in *T. reesei*.

Although molecular genetic manipulation has been applied to the production of most industrial enzymes, lignocellulolytic enzyme systems are primarily produced by fungi that are enhanced through strain improvement^23,24^. The most widely used commercial lignocellulolytic enzymes are produced by *T. reesei* strains that have been improved through repeated mutagenesis and screening processes^25^. Reconstructions of genes, such as the carbon repressor gene *cre1*^26^, β-glucosidase regulator gene *bglR*^18^, glucosidase IIa subunit gene *gls2a*^27^, and the central regulator gene *xyr1*, were found to contribute to the hyperproduction of cellulolytic enzymes in *T. reesei* mutant strains. Mutations in *cre1* and *bglR* lead to relieve CCR^18,28^, the mutated *gls2a* results in altered *N*-glycan patterns on secreted proteins^27^, and a single point mutation in XYR1 is responsible for a highly elevated basal level of cellulase expression^14^. Although a large number of genetic mutations have been identified in several *T. reesei* mutants through comparative genomic analyses^14,29–31^, additional mechanisms accounting for the cellulase-hyperproducing phenotype remain enigmatic. However, these screened mutant genes offer an efficient and rapid method to further improve industrial strains^14,32^.

Under cellulase-inducing conditions, *T. reesei* usually produces and secrets cellobiohydrolases and endoglucanases, comprising approximately 85% and 15% of the total proteins, respectively. By contrast, the proportions of secreted β-glucosidases and hemicellulases are very low^33^. The addition of β-glucosidases or/and xylanases produced by other fungi to *T. reesei* cellulase preparations could increase the enzyme efficiency for hydrolyzing cellulosic substrates^34^. The introduction of heterologous coding genes of β-glucosidases or/and xylanases to the *T. reesei* genome is another method for modifying the enzyme preparation process^33^.

In this study, we found that a putative non-histone methyltransferase (designated *Tr*SAM) in a large lost fragment may be involved in the high β-glucosidase yield of *T. reesei* mutant engineered in our group. We speculated that this methyltransferase may regulate the affinity of ACE1 to promoter regions of cellulase genes through the interaction between *Tr*SAM and the residue R383 of ACE1 and take part in the accurate regulation of CCR. Knowledge of this functional gene may contribute to find engineering strategies for the design of protein hyperproducers.

## Results

### Deletion of a putative protein methyltransferase (*Tr*SAM) contributed to the increased β-glucosidase production in two mutants

**Fig. 1:**
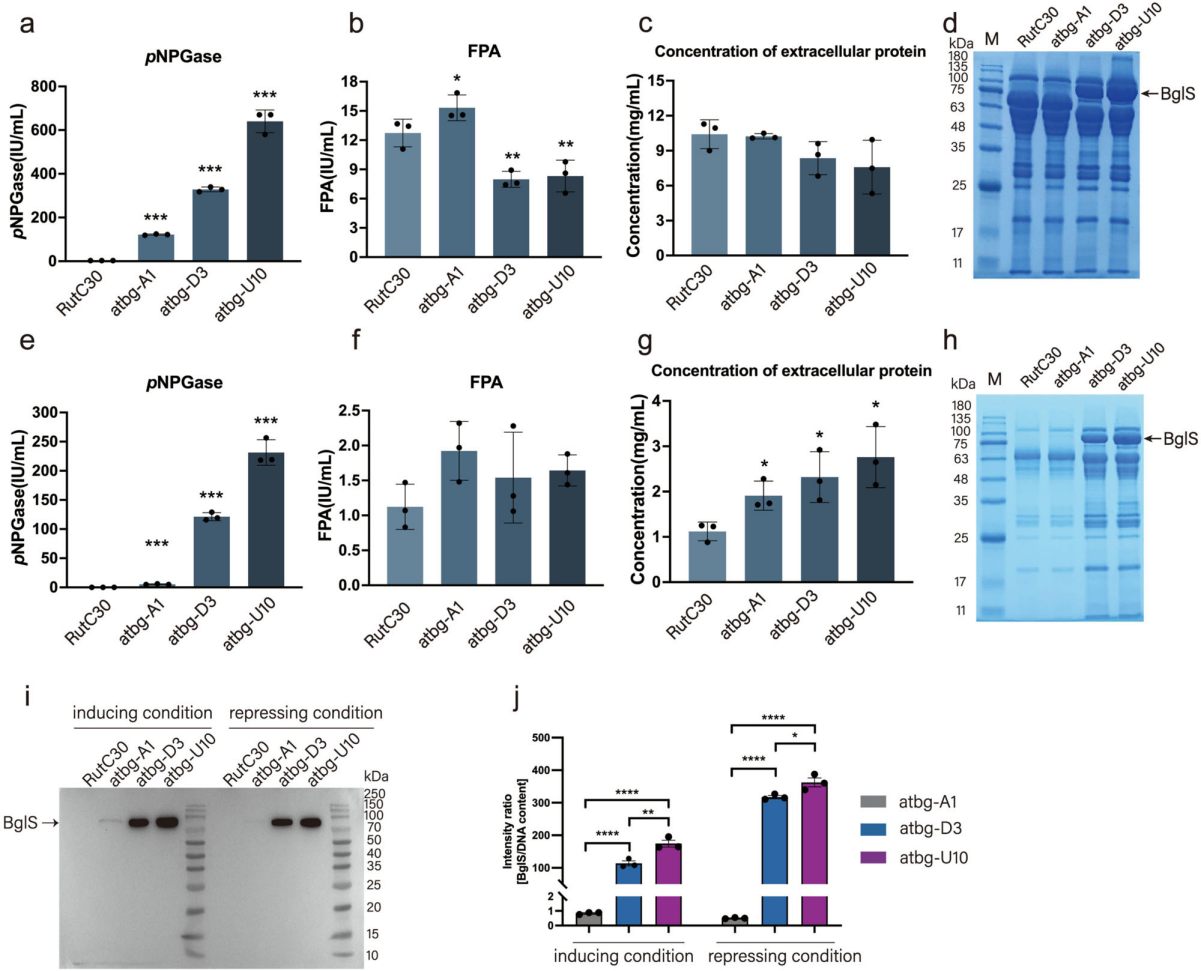
Evaluation of enzymatic activity and protein production by transformants expressing BglS in inducing and repressing conditions. **a** Evaluation of β-glucosidase (*p*NPGase) activities under inducing condition. **b** Evaluation of filter paper activities (FPAs) under inducing condition. **c** Evaluation of extracellular protein concentrations under inducing condition. **d** SDS-PAGE analysis of protein production under inducing condition. **e** Evaluation of *p*NPGase activities under repressing condition. **f** Evaluation of FPAs under repressing condition. **g** Evaluation of extracellular protein concentrations under repressing condition. **h** SDS-PAGE analysis of protein production under repressing condition. **i** Western blot analysis of BglS production, the black arrows indicate BglS. The sample volume of atbg-D3 or atbg-U10 was one-tenth that of atbg-A1. **j** The BglS intensity ratio to DNA content. Inducing condition: 3% Avicel and 2% wheat bran were used as carbon source. Repressing condition: inducing culture added with 2% Glucose. All of the samples were collected after 5-day fermentation. All the samples were tested by three replicates. Data are represented as mean ± SD. *P < 0.05, **P < 0.01, ***P < 0.001. The molecular weight of BglS is 78.49 kDa.

In our previous research, a series of transformants were successfully constructed by the random insertion of the encoding gene of a β-glucosidase from *Aspergillus terreus* (named as *bgls*)^35^, most of them (represented by the mutant atbg-A1) demonstrated significantly increased β-glucosidase production (hydrolysis activity of *p*-nitrophenyl-β-D-glucopyranoside, *p*NPGase activity) and slightly increased filter paper activity (FPA), compared to that of the parent strain Rut-C30. However, one mutant, named as atbg-D3, unexpectedly exhibited lower filter paper activity (60% of that of Rut-C30) but much higher *p*NPGase activity (>100-fold increase) (Supplementary Fig. 1). Subsequently, an orotate-phosphoribosyl transferase (URA5)-disrupted mutant, designated as atbg-U10, was created by the ultraviolet (UV) mutagenesis of atbg-D3 and screened by the highest β-glucosidase activity in this study. Unexpectedly, atbg-U10 exhibited the highest β-glucosidase activity in inducing medium, with a 200-fold increase in *p*NPGase activity compared to Rut-C30 (Fig. 1a).

These strains showed distinct cellulase producing phenotype in response to induction and repression. In inducing medium (induced by 3% Avicel and 2% wheat bran), the *p*NPGase activities of Rut-C30, atbg-A1, atbg-D3 and atbg-U10 were 3.03 ± 0.18, 121.87 ± 2.83, 328.43 ± 10.98, and 639.98 ± 52.05 IU/ml, respectively (Fig. 1a). On the contrary, atbg-D3 and atbg-U10 showed lower FPAs under the same inducing condition (Fig. 1b), despite their extracellular protein concentration did not demonstrate significant difference compared with Rut-C30 (*P* = 0.072638) and atbg-A1 (*P* = 0.070732) (Fig. 1c). These results corresponded to the expression level of BglS and CBH1 in these strains via sodium dodecyl sulfate-polyacrylamide gel electrophoresis (SDS-PAGE) (Fig. 1d). In repressing medium (inducing medium with 2% glucose), the *p*NPGase activities of atbg-A1, atbg-D3 and atbg-U10 were 5.36 ± 0.69, 121.32 ± 6.87, and 231.32 ± 21.85 IU/mL, respectively, and the *p*NPGase activity of Rut-C30 was barely detectable (Fig. 1e). The FPAs of atbg-D3 and atbg-U10 showed no significant difference with that of Rut-C30 (*P* = 0.146768) and atbg-A1 (*P* = 0.316371) (Fig. 1f), whereas their extracellular protein concentration obviously increased compared with Rut-C30 and atbg-A1 under the repressing condition (Fig. 1g, h). The expression of BglS in atbg-D3 and atbg-U10 was still abundant by western blot verification, but such a phenomenon wasn’t observed by atbg-A1 in repressing medium (Fig. 1i, j), which demonstrated that BglS could be expressed when glucose was used as the carbon source in atbg-D3 and atbg-U10 strains. Compared to Rut-C30 and atbg-A1, atbg-D3 and atbg-U10 exhibited more tolerance to glucose repression (Fig. 1h). Thus, we dedicate to analyze the underlying mechanism involved in hyperproduction of *p*NPGase in atbg-D3 and atbg-U10.

**Fig. 2:**
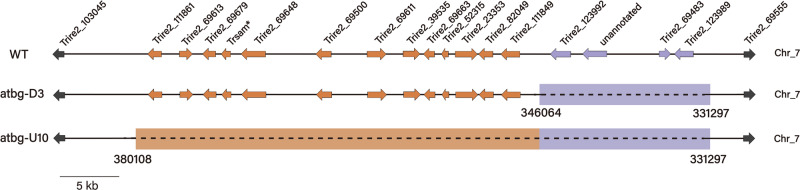
Schematic diagram of large missing fragments in atbg-D3 and atbg-U10. The purple segment represents the lost 14.8-kb fragment in atbg-D3. The orange segment represents the additional lost 34-kb fragment in the mutant strain atbg-U10. The chromosome structure is based on the latest Qm6a reference genome sequence published by the U.S. Department of Energy Joint Genome Institute (https://mycocosm.jgi.doe.gov/pages/blast-query.jsf?db=Trire2). *: *trsam* was incorrectly annotated in the published genome database of *T. reesei*.

Genome walking assay confirmed that a 14.8-kb region adjacent to *cbh1* was lost in the atbg-D3 genome (Fig. 2 and Table 1). In total, four genes including *cbh1* were deleted from the atbg-D3 genome, with reference to the complete genome sequence of *T. reesei* (https://mycocosm.jgi.doe.gov/pages/blast-query.jsf?db=Trire2)^36^. Four deletion transformants of Rut-C30 were built by knocking out the four respective genes lost in the mutant atbg-D3. Except for *cbh1*, the deletion of the other three genes (encoding a swollenin, a palmitoyltransferase domain-containing protein, and an unknown protein) did not affect their FPAs (Table 1). The decreased FPA and higher *p*NPGase activity in atbg-D3 may be mainly resulted from the absence of *cbh1*, which encodes the most abundant extracellular protein, CBH1. The reduction of secretion pressure caused by the loss of *cbh1* increases the secretion of heterologous BglS, which is controlled by the *cbh1* promoter.

**Table 1:** Effect on FPA after deletion of genes in the lost fragment

Gene No.	Annotations of the lost genes	atbg-D3	atbg-U10	Effect on FPA
123989	GH7 Cellobiohydrolase CBH1/CEL7^a^	-^a^	-	Decreased
69483	unknown protein	-	-	NS^c^
-	Palmitoyltransferases domain-containing protein	-	-	NS
123992	Swollenin	-	-	NS
111849	Xylanase 4	+^b^	-	NS
82049	unknown protein	+	-	NS
23353	Ferric reductase	+	-	NS
52315	Copper transporter	+	-	NS
69663	Esterase/lipase/thioesterase	+	-	NS
39535	mannitol 1-phosphate dehydrogenase	+	-	NS
69611	MFS transporter	+	-	NS
69500	Integral membrane protein	+	-	NS
69648	p450	+	-	NS
trsam	methyltransferase	+	-	Increased
69679	unknown protein	+	-	NS
69613	Protein of unknown function DUF227	+	-	NS
111861	hypothetical GPCR	+	-	NS
	Ura5 (selection marker)	+	Mutated	NS
	*hph* (selection marker)	+	-	NS

^a^The minus sign means the gene is lost in the corresponding strain.

bThe plus sign means the gene is present in the corresponding strain.

^c^NS means there is no significant difference between parent and deletion strain.

Considering that atbg-U10 displayed more tolerant to the glucose repression (Fig. 1e, h), genome walking assay was also performed in atbg-U10. According to the sequencing data, another 34.0-kb region adjacent to the previously lost 14.8-kb region in atbg-D3 was missing from atbg-U10 genome. The newly lost region includes 13 annotated genes (Fig. 2). To identify any critical genes affecting cellulase synthesis in this lost region, all 13 genes were respectively deleted in Rut-C30 using CRISPR/Cas9 system. After 3 days of induction, the deletion of *trsam*, annotated as a methyltransferase, increased the FPA of the selected transformants, whereas the FPAs of the other deletion mutants were not affected under the same condition (Table 1). This confirmed that the deletion of *tr*sam contributed to the increase of *p*NPGase activity in atbg-U10.

When the methyltransferase-encoding gene *trsam* was also deleted in *T. reesei* QM6a and its derivatives (QM9414 and Rut-C30) via *Agrobacterium*-mediated transformation, the FPAs of the respective deletion mutants increased by 154%, 108%, and 32%, respectively (Supplementary Fig. 2a). We also employed a heterologous feruloyl esterase encoding gene (*fea*) to verify the effect on protein production of *trsam*. The deletion of *trsam* in the hrFEA strain (with *fea* replacing *cbh1*) could further improved the *p*NPFase activity (hydrolysis activity of *p*-nitrophenol ferulate), which showed no significant difference to aFEA strain (heterologously expressed *fea* in atbg-U10) (*P* = 0.788891) (Supplementary Fig. 2b, c). All the results suggested that the methyltransferase *Tr*SAM plays important roles in the regulation of cellulase synthesis in these strains.

### Interaction with the potential methylation site R383 of ACE1 is indispensable for the *Tr*SAM to take regulatory role on cellulase synthesis in *T. reesei*

To better understand the characteristics of *Tr*SAM, a phylogenetic tree was constructed to analyze the evolutionary interrelationships in a group of fungi. *Tr*SAM was more closely related to an ortholog from the entomopathogenic fungus *Metarhizium robertsii* than to that from another lignocellulolytic fungus, *Penicillium oxalicum* (Supplementary Fig. 3). To investigate the function of *Tr*SAM in the regulation of cellulase production, interactions between *Tr*SAM and widely known cellulase-related regulators were analyzed using the yeast two-hybrid system.

**Fig. 3:**
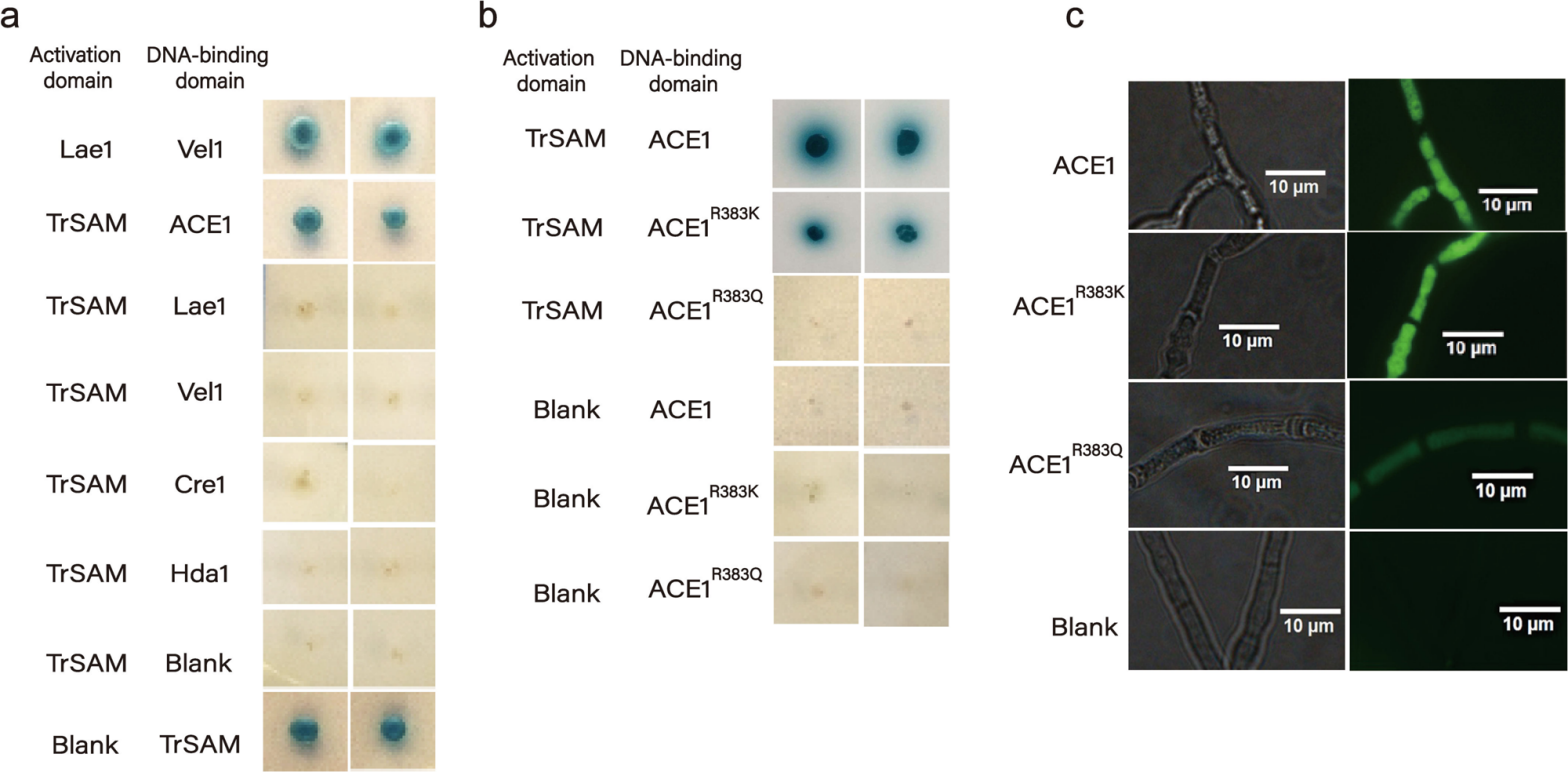
Interaction analysis of protein methyltransferase *Tr*SAM and ACE1. **a** In vitro interaction analysis by yeast two-hybrid assay for screening the interactive regulators of *Tr*SAM. Lae1 (AFX86442.1), Vel1 (OTA08068.1), Cre1 (AAB01677.1), Hda1 (XP_006968036.1). Lae1-AD and Vel1-BD were used as the positive control. Blank for activation domain: pGADT7, blank for DNA-binding domain: pGBKT7. **b** In vitro interaction analysis of ACE1 mutants and *Tr*SAM by yeast two-hybrid assay. **c** In vivo interaction analysis of ACE1 mutants and *Tr*SAM by Bimolecular Fuorescence Complementation assay in 2% glucose repressing medium. Scale bars, 10 μm.

The interaction between *Tr*SAM-AD (*Tr*SAM fused with the GAL4 activation domain) and ACE1-BD (ACE1 fused with the GAL4 DNA-binding domain) promoted yeast growth in the absence of histidine and adenine and resulted in X-α-gal activity, whereas the interaction between *Tr*SAM-AD and Lae1-BD or other fused regulators (Vib1-BD, Cre1-BD, and Hda1-BD) did not (Fig. 3a). These data suggest a possible interaction between *Tr*SAM and the negative regulator ACE1 (Protein ID: XP_006962963.1). According to the Methyl-group Specific Predictor online tool (http://msp.biocuckoo.org/), R59, R226, and R383 in ACE1 are putative methylation residues (Supplementary Table 1). To determine whether the methylation of these sites might take effects on cellulase synthesis, we mutated arginine to glutamine, which presented in much lower methylation abundance, at these specific sites to construct ACE1^R59Q^, ACE1^R226Q^ and ACE1^R383Q^ variants and tested the FPAs of these transformants, respectively. Mutants with ACE1^R383Q^ revealed significantly increased FPAs, contrarily mutants with ACE1^R59Q^ and ACE1^R226Q^ showed similar FPAs as the parent strain Rut-C30 (Supplementary Fig. 4). This result suggests that R383 may be a potential methylation site. We then tried to verify the protein–protein interactions involving *Tr*SAM using the constructed ACE1^R383K^, which could be methylated in detectable abundance, and ACE1^R383Q^ mutants. The yeast two-hybrid system revealed that *Tr*SAM-AD interacted with ACE1^R383K^-BD but not with ACE1^R383Q^-BD (Fig. 3b), implying that the methyltransferase *Tr*SAM may interact with ACE1 by the residue R383. In addition, we performed GST pull-down assay as a reinforcement of yeast two-hybrid results (Supplementary Fig. 5).

We further verified the interactions indicated by yeast two-hybrid analysis via an in vivo approach of bimolecular fluorescence complementation (BiFC). ACE1 and its variants were fused with the C-terminal of enhanced green fluorescent protein (eGFP), and *Tr*SAM was fused with the N-terminal of eGFP. Each pair of the expression cassettes were co-transformed into *T. reesei* 1D4-6 (a uridine-auxotrophic derivative of Rut-C30), respectively. The results showed that ACE1 or ACE1^R383K^ interacted obviously with *Tr*SAM when the transformants were cultured in 2% glucose repressing medium. By contrast, the transformants derived from co-transformation of ACE1^R383Q^ and *Tr*SAM fused with eGFP fragments only produced very weak fluorescence (Fig. 3c) under the same condition. As a blank control, the parent strain without BiFC plasmids did not produce fluorescence. Therefore, we confirmed the direct in vivo interaction between *Tr*SAM and ACE1 or ACE1^R383K^ under repressing condition. Furthermore, the direct in vivo interaction between *Tr*SAM and ACE1^R383Q^ became weaker, possibly because *Tr*SAM could not interact with the mutated Q383 residue in ACE1. When these strains were induced by lactose, no fluorescence was observed in the strain harboring *Tr*SAM and ACE1^R383Q^; meanwhile, a very weak interaction was detected between *Tr*SAM and ACE1 or ACE1^R383K^ (Supplementary Fig. 6). Taking consideration of that *Tr*SAM is more likely to interact with the R383 residue of ACE1 in repressing culture, it suggests that *Tr*SAM played an important regulatory role during CCR in *T. reesei*. After the interactional verification of *Tr*SAM and ACE1 or the mutated ACE1 by in vivo and in vitro assays, we concluded that the putative methyltransferase *Tr*SAM regulated cellulase production likely by the interaction with ACE1 at R383, especially under CCR condition.

### The amino acid residue R383 is crucial for ACE1 to bind the promoters of its target genes and thus results in the repression of cellulase gene expression

**Fig. 4:**
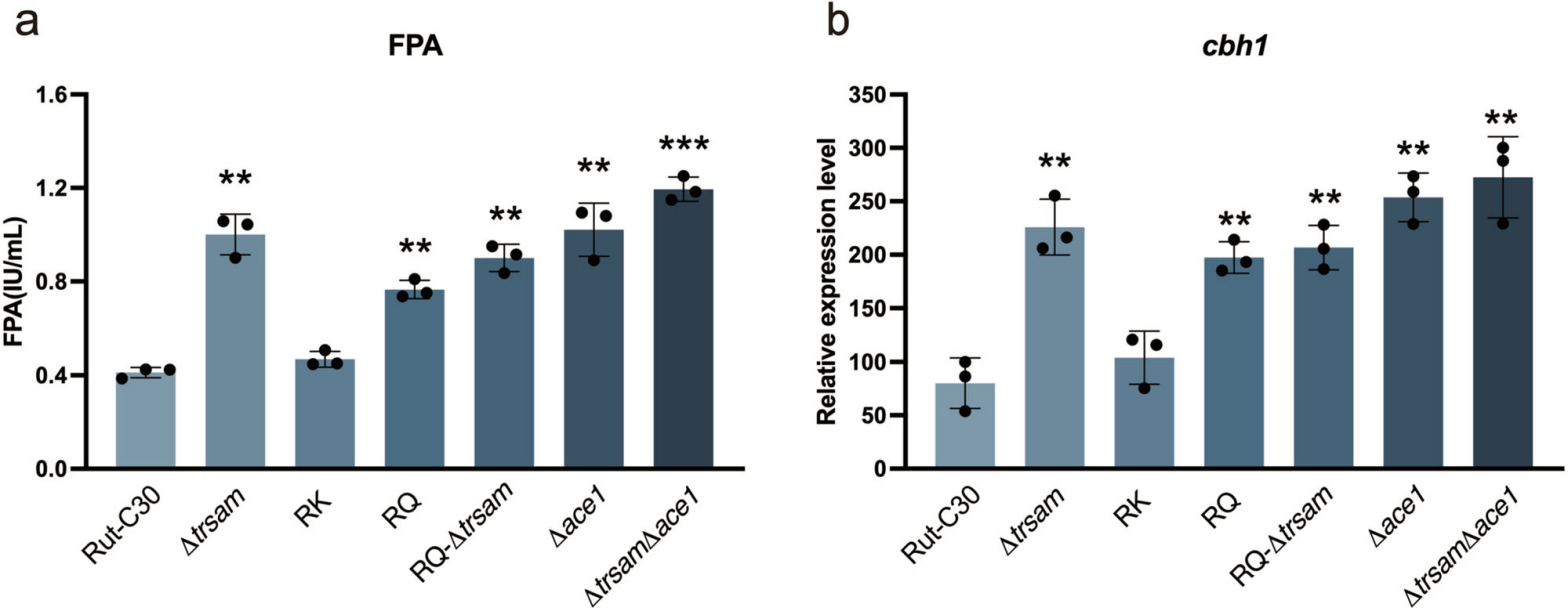
Evaluation of cellulase production of Rut-C30 and its mutants. **a** FPAs of Rut-C30 and its transformants determined after differential deletion of *Tr*SAM and ACE1 in 1% lactose inducing medium. **b** Relative expression levels of *cbh1* in Rut-C30 and its transformants upon induction by 1% lactose. Rut-C30: parent strain. *∆trsam*: *trsam* deletion strain. RK: strain harboring ACE1^R383K^. RQ: strain harboring ACE1^R383Q^. RQ-*∆trsam*: strain harboring ACE1^R383Q^ and deletion of *trsam*. *∆ace1*: *ace1* deletion strain. *∆trsam∆ace1*: *trsam* and *ace1* double deletion strain. All the samples were tested by three replicates. Data are represented as mean ± SD. **P < 0.01, ***P < 0.001.

To further explore the mechanism involved in the phenomenon that *Tr*SAM regulated cellulase expression, which was potentially related to ACE1, we tested the effects of different ACE1 mutations on enzyme activity. The FPA of the mutant strain RQ (harboring the ACE1^R383Q^ mutation) cultured in 1% lactose inducing medium increased by 85.8% compared to the parent strain (*P* = 0.0002) (Fig. 4a). By contrast, the FPA of the mutant strain RK (harboring the ACE1^R383K^ mutation) did not differ significantly from that of the parent strain (*P* = 0.0700) (Fig. 4a). To investigate the relationship between ACE1^R383^ and *Tr*SAM, we further constructed a series of *T. reesei* mutants—the *ace1* deletion strain Δ*ace1*, the *trsam* and *ace1* double deletion strain Δ*trsam*Δ*ace1*, the complemented strain re*trsam*, and a *trsam* deletion strain harboring the ACE1^R383Q^ mutation named RQ-Δ*trsam*—and tested their cellulase activities. All of the tested mutants exhibited markedly increased FPAs except for strain RK in which FPA was not significantly different from the parent strain (*P* = 0.0700) (Fig. 4a). Among these strains, RQ and RQ-Δ*trsam* mutants increased by 85.8% and 118.0% in FPAs, respectively. Although the cellulase activity in the Δ*trsam*Δ*ace1* mutant (189.8%) was not significantly different from that of Δ*ace1* (148.0%) (*P* = 0.0745) and Δ*trsam* (149.4%) (*P* = 0.0764), the Δ*trsam*Δ*ace1* mutant tended to be benefit to cellulase production. When detecting the relative expression levels of *cbh1*, the double deletion strain Δ*trsam*Δ*ace1* had the highest expression level among all tested strains, consistent with their variation in enzymatic activity (Fig. 4b). When the mutant strains RK, RQ, Δ*ace1*, Δ*trsam*, RQ-Δ*trsam* and Δ*trsam*Δ*ace1* cultured in 1% glucose repressing medium, we found that RQ mutant increased by 8.6% in CMCase activity, whereas RK mutant revealed almost unchanged CMCase activity relative to Rut-C30 (*P* = 0.2997). Compared with single amino acid site mutation, gene deletion affected CMCase activity more markedly. RQ-Δ*trsam* and Δ*ace1* mutants increased by 38.3% and 52.7% in CMCase activities, respectively. The Δ*trsam* mutant showed the highest CMCase activity (170.3%), followed by Δ*trsam*Δ*ace1* mutant strain (165.3%), which implying that *trsam* or *ace1* mutation could observably relieve CCR (Supplementary Fig. 7). These above results suggest that *Tr*SAM may take part in CCR pathway through the interaction with ACE1 at R383. It seems that the deletion of *ace1* improved more cellulase production in inducing medium, but the deletion of *Tr*SAM brought more efficient cellulase production under repressing condition. The methylation of ACE1 R383 was not detected using antibodies (Supplementary Figs. 8 and 9a), perhaps due to transient methylation, which is observed in other TFs^37^. In addition, it may also be related to the lower expression of ACE1. However, several arginine residues within the recombinant Glutathione-S-transferase (GST)-ACE1^317-463aa^ were identified as methylation sites, including R383 (Supplementary Fig. 9b). This finding suggests that R383 could be methylated, of course, the specific mechanism underlying this sidechain methylation in relation to cellulase production remains unknown.

**Fig. 5:**
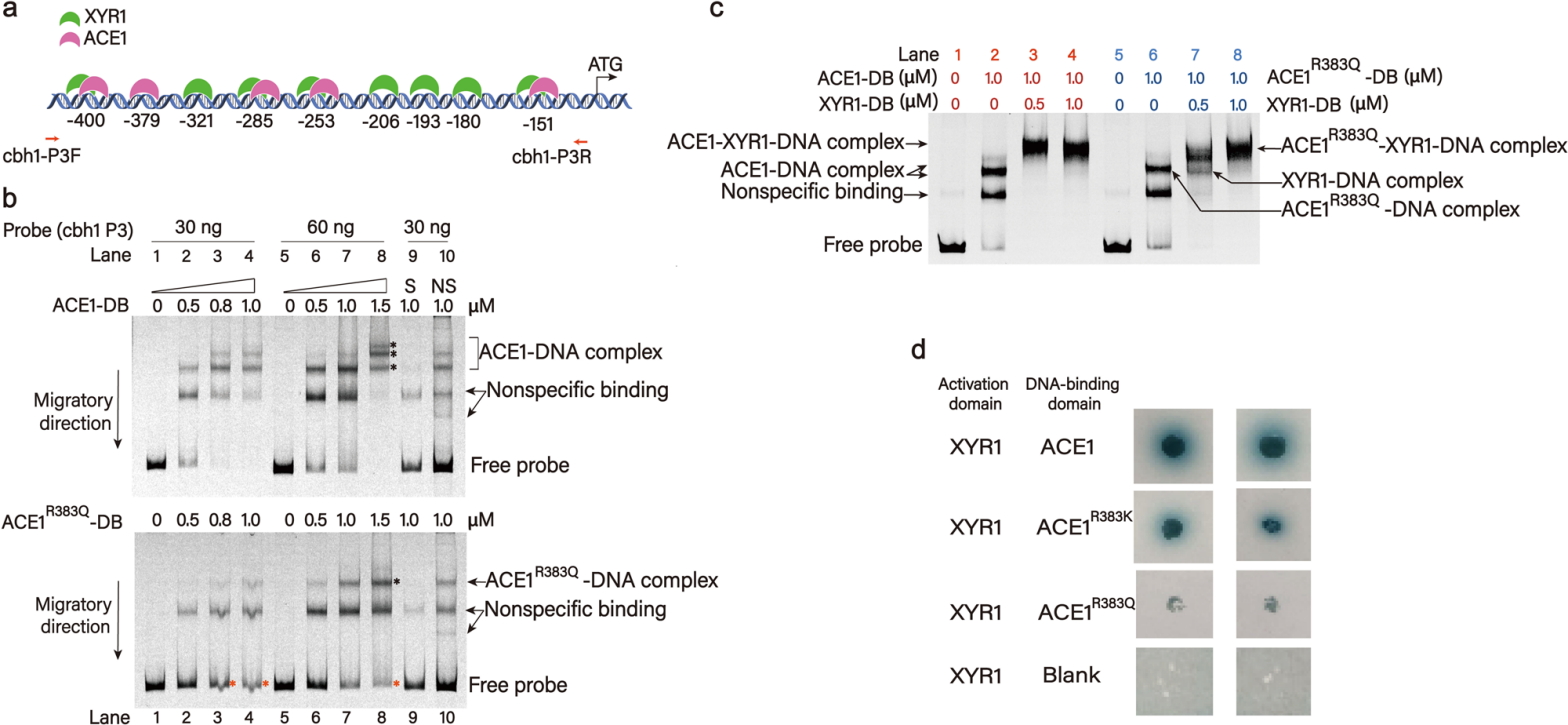
Comparison of ACE1 and ACE1^R383Q^ abilities to bind DNA. **a** Schematic diagram of the binding consensus sequences of ACE1 and XYR1 in the upstream region of *cbh1* (*cbh1*-P3) in *T. reesei*. The locations of the ACE1- and XYR1-binding sites in this promoter region are illustrated. **b** Comparative analysis of *cbh1* P3 binding ability of recombinant ACE1-DB (the upper panel) and the ACE1^R383Q^-DB (the lower panel) by electrophoretic mobility shift assay. Red asterisks: free probes. Black asterisks: ACE1-DB-DNA complex (the upper panel) and ACE1^R383Q^-DB-DNA complex (the lower panel). **c** Evaluation of the competitive *cbh1* P3 binding ability between recombinant ACE1-DB (or ACE1^R383Q^-DB) and XYR1. **d** Comparative analysis of the interactions between XYR1 and ACE1 as well as ACE1 mutants by yeast two-hybrid assay.

XYR1 is the most critical cellulase activator in *T. reesei*. It has been confirmed that ACE1 antagonizes *xyr1* transcription by binding to the promoter regions of *xyr1*^38^ and/or cellulase genes^39^ to downregulate the expression of cellulases, competing with XYR1 for certain binding sites. To confirm the DNA-binding capacity of ACE1 and its mutants, electrophoretic mobility shift assay (EMSA) were performed using the *cbh1* promoter and the DNA-binding domain of ACE1 (ACE1-DB, 317−528 aa), which included residue R383. GST-tagged ACE1-DB and ACE1^R383Q^-DB recombinant proteins were purified from *E. coli*. The probe sequence (designated *cbh1*-P3) extended from nucleotide position −79 to −420 of the corresponding upstream coding region of *cbh1*, which contained five ACE1 binding consensus sequences [5’-(A)GGCA-3’] (−151, −253, −285, −379, and −400) and eight XYR1 binding consensus sequences [5’-GGC(A/T)_3_−3’] (−151, −180, −193, −206, −253, −285, −321, and −400). Four ACE1 sites coincide with XYR1 sites at nucleotide positions −151, −253, −285, and −400 (Fig. 5a). According to the EMSA results, typical protein concentration-dependent binding was observed. More ACE1-DB–DNA complex was observed than ACE1^R383Q^-DB–DNA complex (Fig. 5b, lanes 2, 7, and 8). In addition, 1.5 μM ACE1-DB could completely bind 60 ng of *cbh1*-P3 probe, whereas ACE1^R383Q^-DB could not (Fig. 5b, lanes 4 and 8), indicating that ACE1 had a stronger ability to bind to *cbh1*-P3 than the mutant ACE1^R383Q^.

In *cbh1*-P3, there were eight XYR1 binding sites, four of which coincided with ACE1 binding sites. We suspected that ACE1 and ACE1^R383Q^ had different abilities to form protein–DNA complexes. The ACE1-DB and XYR1-DB recombinant proteins were analyzed together or separately in EMSA with the *cbh1*-P3 probe. When applied separately, both ACE1-DB and ACE1^R383Q^-DB bound to Cy5-labeled *cbh1*-P3, forming two distinct protein–DNA complexes (Fig. 5c, lanes 2 and 6). By contrast, when 0.5 μM XYR1-DB (55−195 aa) was then added, all ACE1-DB participated in the formation of the two proteins–DNA complexes, whereas the mutant ACE1^R383Q^-DB did not (Fig. 5c, lanes 3 and 7). XYR1-DB exhibited stronger competitive binding ability with *cbh1*-P3 when it was incubated together with ACE1^R383Q^-DB as the specific XYR1–DNA complex was more distinguish in lane 7 of Fig. 5c. In order to explain whether this difference was caused by the change of interaction between XYR1 and ACE1 before and after the R383 mutation, we also assayed the ACE1, ACE1^R383K^ and ACE1^R383Q^ variants by yeast two-hybrid to verify their protein–protein interactions with XYR1. The Y2H results showed that interactions existed between XYR1-AD and ACE1-BD or ACE1^R383K^-BD, but not between XYR1-AD and ACE1^R383Q^-BD, which were consistent with the results of the EMSA experiment (Fig. 5c, d).

**Fig. 6:**
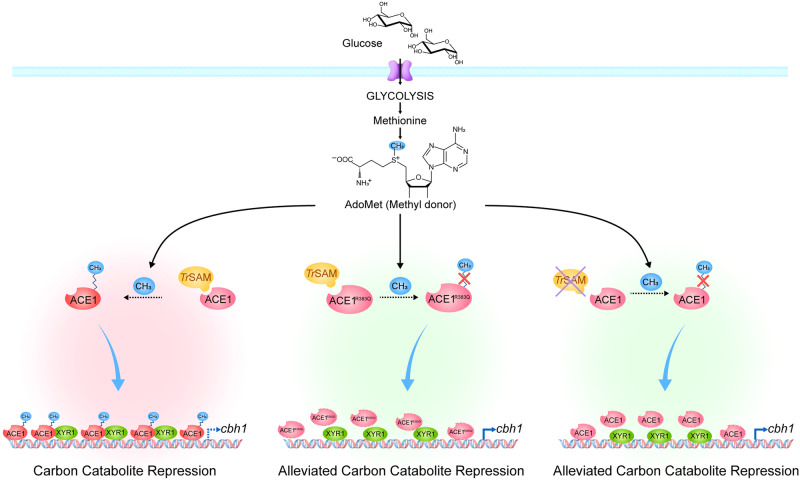
Schematic model of *Tr*SAM-mediated regulatory mechanism during carbon catabolite repression in *T. reesei.* The putative methyltransferase *Tr*SAM interacts with the transcription repressor ACE1 and methylates it at potential methylation site R383 in glucose medium, which activates ACE1 to compete with XYR1 for binding cellulase genes promoter thus results in CCR (left panel). When ACE1 R383 residue is mutated to glutamine (middle panel) or *Tr*SAM is knocked out (right panel), ACE1 is inactivated which led to impaired DNA binding ability and thus relieves CCR under repressing condition in *T. reesei*.

These experiments confirmed that the ability of mutated ACE1^R383Q^ to bind the *cbh1* promoter was weakened obviously, and the competitive capacity of ACE1^R383Q^-DB against XYR1-DB was reduced compared with that of ACE1-DB, indicating that the DNA-binding ability of ACE1 may be impaired by the mutation of residue R383. Therefore, the R383 may play a crucial role in maintaining the function of ACE1, i.e., repressing cellulase gene expression (Figs. 4 and 6). On the other hand, the de-repressing effect was stronger in strains with ACE1 or *Tr*SAM completely knocked out, indicating that ACE1 retained its part regulatory effect after the mutation of R383. It seems that the interaction between *Tr*SAM and ACE1 at R383 residue may boost its binding to the promotor regions of target genes and thus take parts in CCR regulation pathway (Fig. 6).

## Discussion

Carbon catabolite repression (CCR) is a common phenomenon in microorganisms to be enable efficient utilization of carbon nutrients. In *Ascomycota*, it has been proved that CCR is triggered by Mig1/2 or the homologs Cre1/CreA. In *Basidiomycota*, Roc1 was identified as a transcriptional factor related to CCR. Given the early emergence of Roc1, its origin may precede the emergence of efficient wood decay systems some ~300 million years ago. In *T. reesei*, the negative regulator Cre1 was demonstrated to mediate CCR by the de-repression phenomenon in Cre1-truncated strain. However, it is still obscure whether there is the synergy between Cre1 and repressors, including ACE1, as well as the de-repression of CCR caused by activators, such as XYR1, ACE2, ACE3, and others^5^. C2H2-type TF ACE1 was identified as a repressor of cellulase and xylanase expression in *T. reesei*. It has been testified that ACE1 and XYR1 may bind to the promoters of *xyr1*, *cbh1* and other cellulase or hemicellulase genes competitively^5^.

In this study, we found that the methyltransferase *Tr*SAM participates in the repression of cellulase synthesis by the interaction with the negative regulator ACE1 and thus strengthening its binding to the promotor regions of target genes (Fig. 5). By contrast, the *Tr*SAM deletion mutant or the mutant ACE1^R383Q^, which interacted in much lower abundance, could alleviate CCR under glucose repression (Supplementary Fig. 7). It seems that this novel regulatory pathway of the interaction between *Tr*SAM and ACE1 could supplement CCR mechanism, which was responsible for the elaborate and intricate regulatory network on cellulase synthesis. Although it is possible that the low expression level of ACE1 under inducing condition has weakened its interaction with *Tr*SAM, which further impairs its repressing effect on cellulase expression, the mutation of 383 residue arginine to glutamine or deletion of *trsam* has been demonstrated to improve cellulase production regardless of inducing or repressing conditions (Fig. 4 and Supplementary Fig. 7), and we speculate that this improvement is due to the un-happened interaction. According to a previous report, the cellulase repressor ACE1 forms two proteins–DNA complexes with the activator XYR1 on the promoters of cellulases and related regulators in repressing condition, whereas another XYR1 competes with and replaces ACE1 in inducing condition^40^. Our results confirmed that ACE1 and XYR1 could competitively combine with *cbh1* P3 to form two proteins–DNA complexes, while the mutated ACE1^R383Q^ showed the reduced competitiveness under the same condition (Fig. 5c). Although methylation at R383 has not been detected by various means (Supplementary Figs . 8, 9a and Supplementary Table 2), all the data in this study provide strong evidence that R383 plays an important role not only in the interaction of ACE1 and *Tr*SAM, but also in keeping intact DNA binding affinity of the transcriptional inhibitor ACE1 (Figs. 3 and 5).

In yeast, glycolysis supplies S-adenosylmethionine (S-AdoMet) for methyltransferase to catalyze methylation reactions^41^. And this glycolysis pathway has been confirmed to be involved in glucose-induced CCR in *T. reesei*. The disruption of glycolysis enhanced cellulase production in glucose-containing medium^12^. We speculated that the enhanced glycolysis in repressing medium containing glucose supplies sufficient S-AdoMet for catalyzing the methylation of ACE1 by *Tr*SAM to activate its negative regulation of cellulase synthesis (Fig. 6). After the deletion of *trsam*, the negative regulation by ACE1 of cellulase production via binding to the promoter regions of *xyr*1 and cellulase genes also decreased. Taken together, the results in this study imply a comprehensive novel regulatory model for cellulase synthesis, with the interaction between ACE1 and *Tr*SAM in the presence of glucose potentially playing a crucial role in maintaining the repressive function of ACE1 in cellulase gene expression. However, the methylation of ACE1 could not be verified, and further research is necessary to support this regulatory model.

Protein methylation modification is widely distributed in eukaryotes, and most known methyltransferases specifically catalyze the methylation modification of histones. However, methyltransferases with non-histone proteins as substrates are increasingly reported recently. The important tumor suppressor p53, is one of the models for studying non-histone lysine methylation modifications^42–44^. In addition, the mammal-derived protein methyltransferase complex PRC2 methylates the histone H3K27 site as well as the transcription factor GATA4 at K299 residue impairing its transcriptional activity^45^. Furthermore, in the process of differentiation of animal muscle cells, lysine methyltransferase G9a interacts with transcription factor MyoD, and methylates K104 residue of MyoD, thereby reducing its transcriptional activity^46^. These results revealed that methylation of transcription factors is important for regulating their transcriptional activities. However, there are few researches about the methyltransferases with transcription factors as substrates in filamentous fungi.

The methyltransferase Lae1 is widely regarded as a global regulator and has an important effect on secondary metabolism in *T. reesei*^19^. Moreover, Lae1 is also essential for cellulase production and sporulation in *T. reesei*, and the expression of the general cellulase regulator XYR1 is Lae1-dependent. Thus, Lae1 is a positive regulator of the biosynthesis of secretory proteins in *T. reesei*. The regulatory mechanisms of Lae1 remain to be unknown^47^, because the histone methylation level was not altered by the deletion of *lae1*, and no other proteins have been identified as methylated by Lae1. The deletion of *trsam* increased the production of secretory proteins indicates that *Tr*SAM is a negative regulator of cellulase production in *T. reesei*. And *Tr*SAM differs from Lae1 based on its hypothesized functions in this study.

As a methyltransferase belonging to the Methyltransf_25 family (Pfam ID: PF13649), *Tr*SAM is a small methyltransferase using S-AdoMet as a donor for methyl transfer, with diverse substrate specificities. Protein interaction assays suggest that *Tr*SAM is involved in controlling the transcription of cellulase genes via interacting the negative regulator ACE1. Post-translational modification of TFs has also been demonstrated to be important in transcription regulation^48^. However, *Tr*SAM differs from other protein methyltransferases, including protein arginine methyltransferase (PRMT) and protein lysine methyltransferase (PKMT), which have been reported in other fungi^49,50^. The *trsam* gene only has one S-AdoMet-dependent methyltransferase domain and is much smaller than other reported protein methyltransferases^51^. In addition, the functions of *trsam* orthologous genes in filamentous fungi have also not been investigated^52,53^.

The phylogenetic tree also showed that *Tr*SAM differed from other well-investigated methyltransferases (Supplementary Fig. 10). Like other proteins modified by PRMT, the protein interaction between ACE1 and *Tr*SAM decreased when R383 of ACE1 was mutated to glutamine^54,55^. In addition, the affinity between ACE1^R383Q^ and the cellulase promoter also decreased. This indicates that interaction between *Tr*SAM and ACE1 mediated by ACE1 R383 is required for its regulatory function, suggesting that *Tr*SAM may be a novel type of PRMT.

## Methods

### Strains and culture conditions

*T. reesei* strains including QM6a (ATCC 13631) and Rut-C30 (ATCC 56765)^56^ were maintained on potato dextrose agar plate (PDA) with uridine at 28 °C for 7 days for spore collection^57^. *E.coli* DH5α and BL21 (DE3) were used as cloning and exprssion hosts respectively, and cultured at 37 °C in Luria-Bertani (LB) medium. *Agrobacterium tumefaciens* AGL1 was used to transform the gene to *T. reesei* strains. To induce cellulase production, the conidial suspension (0.5 mL, 1 × 10^7^ conidia/mL) was inoculated into a 50 mL Erlenmeyer flask containing 10 mL of Sabouraud Dextrose Broth (SDB) and incubated for 30 h on an orbital shaker at 200 rpm at 28 °C. The culture was then transferred into a flask containing 10 mL of inducing fermentation medium at 10% inoculum ratio (v/v). The flasks were incubated on an orbital shaker at 200 rpm at 28 °C for 7 days. The wheat bran and Avicel medium was prepared as follows: 4 g KH_2_PO_4_, 2.8 g (NH_4_)_2_SO_4_, 0.6 g MgSO_4_·7H_2_O, 0.5 g CaCl_2_, 0.6 g urea, 3.0 g tryptone, 0.1% tween-80 (v/v), 5.0 g CaCO_3_, 0.01 g FeSO_4_·7H_2_O, 0.0032 g MnSO_4_·H_2_O, 0.0028 g ZnSO_4_·7H_2_O, 0.004 g CoCl_2_·6H2O, 20.0 g wheat bran, 30.0 g microcrystalline cellulose, in 1.0 L water. We also used minimal medium (MM) containing 5.0 g (NH_4_)_2_SO_4_, 15.0 g KH_2_PO_4_, 0.6 g MgSO_4_·7H2O, 0.6 g CaCl_2_, 0.005 g FeSO_4_·7H_2_O, 0.0016 g MnSO_4_·H_2_O, 0.0014 g ZnSO_4_·7H_2_O, 0.002 g CoCl_2_·6H_2_O, in 1.0 L water, and with 10.0 g lactose or glucose used as carbon source for fermentation. All reagents not specified were purchased from Shanghai Sinopharm.

### Heterologous expression of *bgls*

For overexpression of *bgls* under modified promoter P*cbh1*M2 in *T. reesei* Rut-C30^56^, we fused the P*cbh1*M2, *bgls* coding region and the *trpC* terminator from *Aspergillus nidulans* and inserted this fragment into *Hind*III and *Xba*I sites of pXBthg with ClonExpress II One Step Cloning Kit (Vazyme, Nanjing, China). All vectors constructed were verified by sequencing. *Agrobacterium*-mediated transformation was conducted to express heterologous BglS. Briefly, *A. tumefaciens* AGL1 carrying pXBthg-BglS-His was cultivated in LB medium at 28 °C to reach an OD_660_ value of 0.6−0.8. The Rut-C30 conidia were collected from a PDA plate with ddH_2_O containing 0.02% Tween 80 and 0.85% NaCl. The conidial suspension of *T. reesei* Rut-C30 (10^7^/mL) were then combined with an equal volume of *A. tumefaciens* cells and spread on co-culture plate containing 200 μM acetosyringone. After incubation at 26 °C for 2 days, the cultures were transferred to PDA plates supplemented with 25 μg/mL hygromycin B, serving as a selection agent for positive transformants^33^.

### Heterologous expression of feruloyl esterase

The heterologous feruloyl esterase encoding gene *fea* (Gene symbol: ATEG_08907) was amplified from the cDNA obtained from *Aspergillus terreus* NIH2624 (https://www.ncbi.nlm.nih.gov/datasets/taxonomy/341663/) and fused with the reconstructive promoter P*cbh1*M2^56^ and *trpC* terminator. The expression vector was transformed into *T. reesei* Rut-C30 genome by *Agrobacterium*-mediated fungal transformation for randomly insertion of *fea*. The fusion cassette P*cbh1*M2-*fea*-T*trpC* was inserted into *cbh1* loci and the 48.8 kb fragment lost loci by homologous recombination derived from CRISPR-Cas9, respectively.

### Genome walking and sequencing

Genome walking was carried out for the insertion site of *bgls* in atbg-D3 and atbg-U10 following the introduction of Genome Walking Kit (Takara, Dalian, China). The generated fragments were blasted on JGI to find out the homologous sequence of QM6a.

### Construction deletion library

Strain UPDC (a uridine-dependent strain derived from Rut-C30) were used as host. The gRNA cassette including synthetic gRNA sequence and target DNA of the listed genes (Supplementary Data 1) was derived by T7 promoter and transcribed into RNA in vitro with MEGAscript T7 Kit (Ambion, Austin, TX, USA). Donor DNA contain the 5’- and 3’ flanking sequences of listed gene and the selectable marker cassette (the *ura5* gene from *Penicillium oxalicum* controlled by the *gpda* promoter and *trpC* terminator, P*gpda*-*poura5*-T*trpcC*) was ligated into the pMD-18T vector (Takara, Dalian, China). All the donor DNAs were designed homology arms for recycling *ura5* cassette and excluding ectopic insertion. The gRNA and donor DNA were co-transformed by using a modified polyethyleneglycol-mediated protoplast transformation procedure^58,59^. The transformants were selected using minimal medium plates with 1% glucose as carbon source.

### Protein extraction and Western blot analysis

*T. reesei* mycelia were harvested following a 24-h growth in glucose medium and ground with liquid nitrogen. The pulverized samples were then resuspended in Lysis buffer (50 mM HEPES pH 7.5, 150 mM NaCl, 10% glycerol, 0.02% NP-40 and 1 mM PMSF), followed by centrifugation at 12,000 rpm for 10 min at 4 °C to eliminate cell debris. The resulting supernatant was collected as intracellular proteins. The extracted protein samples were fractionated using SDS-PAGE and subsequently transferred to a cellulose acetate membrane. This membrane was then blocked with 10% skim milk in TBST and incubated with primary antibodies, including the anti His-tag mouse monoclonal antibody (Yeasen, Shanghai, China, #30401ES) at a dilution of 1:5000 and the anti-dimethyl arginine mouse monoclonal antibody (Abcam, #ab413) at a dilution of 1:500. Following to incubation with the primary antibodies, the membrane was washed three times with TBST and then incubated with Peroxidase AffiniPure Goat Anti-Mouse antibody (Yeasen, Shanghai, China, #33201ES). The membrane was washed three times in TBST prior to enhanced chemiluminescence detection (Tanon, Shanghai, China).

### Quantitative analysis of heterogenous BglS

For a quantitative analysis of BglS protein derived from atbg-A1, atbg-D3 and atbg-U10 strains, all of them were cultured in either inducing or repressing medium for 5 days and Rut-C30 strain served as a negative control. For western blot analysis with anti His-tag mouse monoclonal antibody (Yeasen, Shanghai, China, #30401ES), 5 μL of supernatants from Rut-C30 and atbg-A1 fermentation broth were utilized as loading samples, while 0.5 μL of supernatants from the atbg-D3 and atbg-U10 fermentation broth were diluted to a volume of 5 μL to serve as loading samples. Image J software was used to analyze the gray values of BglS protein bands revealed by immunoblotting. The DNA contents within the *T. reesei* mycelia were quantified using diphenylamine-colorimetric method^60^. Briefly, 1.5 g diphenylamine is dissolved in 100 mL of glacial acetic acid, followed by the addition of 1.5 mL sulfuric acid to yield solution A. Solution B comprises 1.6% acetaldehyde. These two solutions are mixed at a ratio of 200:1. Subsequently, 1 mL of this mixture is introduced into each mycelia sample. After a 1-h reaction at 60 °C, the absorbance value of the supernatant within the reaction system is measured at 595 nm using Varioskan Flush (Thermo, American). The ratio of gray value to biomass serves as an indicator of the BglS production capacity of the tested strains. In inducing medium, 3% Avicel and 2% wheat bran were used as carbon sources. Additionally, 2% glucose was incorporated into the inducing medium to serve as the repressing medium.

### In vitro methyltransferase assay

The proteins *Tr*SAM and ACE1^317-463aa^ were individually fused with the GST protein tag. Subsequently, these fusion proteins were introduced into the expression host *E. Coli* BL21 (DE3), followed by an 18-hour induction using 0.1 mM IPTG. The recombinant GST-*Tr*SAM and GST-ACE1^317-463aa^ proteins were purified using Glutathione Beads (Smart-LifeSciences, China) and employed as the methyltransferase (5 μg) and substrate (10 μg) respectively for methylation. S-AdoMet served as the methyl donor. The methylation solutions were incubated in reaction buffer (50 mM Tris-HCl pH 8.5, 20 mM KCl, 10 mM MgCl_2_, 100 mM sucrose and 1 mM β-mercaptoethanol) at 28 °C for specific durations and subsequently fractionated by SDS-PAGE. Western blot analysis was performed using the anti-dimethyl arginine mouse monoclonal antibody (Abcam, #ab413) and Peroxidase AffiniPure Goat Anti-Mouse antibody (Yeasen, Shanghai, China, #33201ES).

### Mass spectrometry analysis for methylation sites

For analysis of protein from *T. reesei* mycelia, the intracellular proteins were extracted as previously described. The His-ACE1^317-463aa^ protein was purified using High Affinity Ni-NTA Resin (GenScript, Nanjing, China) from the intracellular proteins. The purified His-ACE1^317-463aa^ was digested by trypsin whereafter the resulting peptides mixture was analyzed using a Dionex Ultimate 3000 RSLCnano system coupled to a Orbitrap Fusion Lumos mass spectrometer. For analysis of protein from *E. coli*, the GST-ACE1^317-463aa^ protein was purified using Glutathione Beads (Smart-LifeSciences, China) from *E. coli* BL21 (DE3). The desalted His-ACE1^317-463aa^ was digested by trypsin and the peptides were separated using RP-C18 column and subsequently analyzed by Q Exactive HF-X (Thermo Fisher).

### Yeast two-hybrid assay

Matchmaker® Gold Yeast Two-Hybrid System (Takara, Dalian, China) was used to analysis interactions among *Tr*SAM and cellulase-related transcriptional regulators including Lae1, Vel1, Cre1, Hda1 and ACE1. The gene *trsam* or *xyr1* was fused to the GAL4 activation domain in pGADT7 yeast expression vector. All the regulator coding genes were ligated to the GAL4 DNA binding domain (DNA-BD) in the pGBKT7 vector, respectively (Supplementary Data 1). The transformation procedure was according to the instructions of the manufacturer. The transformants were screened on dropout plate with 15 μM/mL 3-amino-1,2,4-triazole (Sigma-Aldrich, St. Louis, MO, USA) and 20 μg/mL X-α-GAL (Sigma-Aldrich, St. Louis, MO, USA).

### Bimolecular Fluorescence Complementation (BiFC) Assay

The N-terminus of GFP was fused to *Tr*SAM and the C-terminus of GFP was fused to ACE1. The fused genes *N-GFP-trsam* and *ace1*-*C-GFP*/*ace1(RK)*-*C-GFP/ace1(RQ)*-*C-GFP* were inserted into binary vectors respectively to construct three plasmids containing N-GFP-*Tr*SAM and ACE1-C-GFP or it mutans. Then we randomly inserted the fragments into the genome of *T. reesei* 1D4-6 (a uridine-auxotrophic derivative of *T. reesei* Rut-C30) by *Agrobacterium*-mediated transformation. The positive transformants were verified by sequencing. And then the conidial suspension was inoculated into a 50 mL flask containing 10 mL of Sabouraud Dextrose Broth (SDB) and incubated for 30 h on an orbital shaker at 200 rpm at 28 °C. The culture was then transferred into a flask containing 10 mL of MM fermentation medium complemented with 2% glucose at 10% inoculum ratio (v/v). The flasks were incubated on an orbital shaker at 200 rpm at 28 °C. After 24 h, took samples from the flasks respectively and observed them by microscope Olympus BX53.

### GST pull down assay

The recombinant GST and GST-ACE1^317-463aa^ were purified using Glutathione Beads (Smart-LifeSciences, China) from *E. coli* BL21 (DE3) following an 18-hour induction with 0.1 mM IPTG. Similarly, the recombinant His-*Tr*SAM protein was purified using High Affinity Ni-NTA Resin (GenScript, Nanjing, China) from *E.coli* under identical condition. A weight of 20 μg purified GST or GST- ACE1^317-463aa^ fusion proteins was incubated with 20 μg His-*Tr*SAM in 500 μL incubation buffer (50 mM Tris-HCl pH6.8, 250 mM NaCl, 1.5% glycerol, 0.6% Triton X-100 and 0.1% Tween-80) for 4 h at 4 °C. The beads were then washed three times with the incubation buffer. The washed beads were boiled in SDS loading buffer and subsequently separated followed by western blot analysis with anti-GST (Yeasen, Shanghai, China, #30901ES) and anti His-tag mouse monoclonal antibody (Yeasen, Shanghai, China, #30401ES).

### Enzyme activity assays

The specific activities of the cellulase system secreted by *T. reesei* on FPase activity were measured using a modified IUPAC method^61^. The assay mixture was incubated at an optimal condition of 60 °C, pH 4.8 for 60 min, and the reaction was stopped with 120 μL DNS followed by an incubation in boiling water for 10 min. The *p*NPGase and CMCase were measured using *p*-Nitrophenyl-β-D-glucopyranoside (*p*NPG) and sodium carboxymethylcellulose (CMC-Na) as substrate, respectively. A unit of enzyme activity (U) was defined as the number of micromoles of reducing sugar or *p*NP released per minute per milligram protein or per milliliter fermented culture. Student’s *t* test was performed with Excel 2007 (Microsoft, WA, USA), employing a two-tailed test. Proteins were quantified using the DC protein assay kit (Bio-Rad, Hercules, CA, USA), according to the manufacturer’s instructions.

### Electrophoretic mobility shift assay

Cy5-labeled DNA probe containing the *cbh1* promoter P3 (−420 ~ −79 relative to the translation start site) was produced by two-step PCR. First, the genomic DNA of *T. reesei* Rut-C30 was used as the template and the non-labeled PCR products were obtained. Secondly, the PCR products were used as the template after purification and the primers using in this step was Cy5-labeled. Then the final PCR products were purified and quantified to acquire the Cy5-labeled probes. The DNA-protein binding assay was achieved by incubating the recombinant ACE1-DNA binding domain (317−528 aa), or the ACE1^R383Q^ DNA binding domain (317−528 aa, containing the mutant of R383Q), with 30 ng or 60 ng of the Cy5-labeled DNA probe in reaction buffer for 20 min at 25 °C. In competitive EMSA, XYR1-DB (55−195 aa), ACE1-DB and Cy5-labeled DNA probe were incubated together. After incubation, the non-denaturing polyacrylamide gel electrophoresis was used to separate protein-bound and free probes. Finally, fluorescence and image analysis of the gels was carried out on Fuji Film FLA-9000 (Fujifilm, Japan).

### Statistics and reproducibility

Three biological replicates were used under each culture condition. Two-tailed *t* tests were employed for statistical analysis at a 95% confidence interval. The source data are deposited in Supplementary Data 2.

### Reporting summary

Further information on research design is available in the Nature Portfolio Reporting Summary linked to this article.

### Supplementary information

Supplementary information

Description of Additional Supplementary Files

Supplementary Data 1

Supplementary Data 2

Reporting Summary

## Data Availability

All data supporting the results of this study are available within the paper and its Supplementary Data. Source images for SDS-PAGE, Western Blot and EMSA are included in Supplementary information (Supplementary Fig. 11-13). The sequencing data of *Tr*SAM has been deposited as GenBank accession number PP390251.
