# Supplementary material for: The protein methyltransferase TrSAM inhibits cellulase gene expression by interacting with the negative regulator ACE1 in Trichoderma reesei
Source: Commun Biol. 2024 Mar 28;7:375. doi: 10.1038/s42003-024-06072-1 (PMC10978942; doi:10.1038/s42003-024-06072-1)
Supplement: Supplementary file 1 — Supplementary information [file 42003_2024_6072_MOESM1_ESM.pdf]

The protein methyltransferase *Tr*SAM inhibits cellulase gene expression by interacting with the negative regulator ACE1 in *Trichoderma reesei*

Zhihua Zhu[1, 2, †], Gen Zou[1, 3, †], Shunxing Chai[1, 2], Meili Xiao[1, 2], Yinmei Wang[1,2], Pingping Wang[1], Zhihua Zhou[1*]

[1] CAS-Key Laboratory of Synthetic Biology, CAS Center for Excellence in Molecular Plant Sciences, Institute of Plant Physiology and Ecology, Chinese Academy of Sciences, 300 FengLin Rd, Shanghai, 200032, China.

[2] University of Chinese Academy of Sciences, Beijing, 100049, China.

[3] Shanghai Key Laboratory of Agricultural Genetics and Breeding, Institute of Edible Fungi, Shanghai Academy of Agriculture Science, 1000 Jinqi Rd, Shanghai, 201403, China.

[†] These authors contributed equally.

*Correspondence: zhouzhihua@cemps.ac.cn

The Supplementary information include:

Supplementary Fig. 1: Evaluation of enzymatic activities and protein concentrations of the cellulase preparation of *T. reesei* Rut-C30 and its BglS transformants.

Supplementary Fig. 2: Evaluation of heterologous protein FEA production in *Tr*SAM deletion strain.

Supplementary Fig. 3: Phylogenetic tree of *Tr*SAM and its fungal orthologues.

Supplementary Fig. 4: Evaluation of enzymatic activities of *T. reesei* and its transformants harboring ACE1 variants induced by 1% lactose.

Supplementary Fig. 5: The interaction between ACE1 and *Tr*SAM was studied by *in vitro* pull-down assay.

Supplementary Fig. 6: Analysis of interaction between *Tr*SAM and ACE1 variants induced by 1% lactose.

Supplementary Fig. 7: CMCases of *T. reesei* Rut-C30 and its mutants in repressing medium.

Supplementary Fig. 8: Detection of ACE1 methylation *in vivo*.

Supplementary Fig. 9: Detection of ACE1 methylation *in vitro*.

Supplementary Fig. 10: Phylogenetic tree of *Tr*SAM and well-investigated arginine methyltransferases in filamentous fungi.

Supplementary Fig. 11: Source gel image of protein production by SDS-PAGE.

Supplementary Fig. 12: Source blot image of BglS production by Western blot.

Supplementary Fig. 13: Source gel image of EMSA analysis.

Supplementary Table 1 Prediction of putative methylation sites of ACE1

Supplementary Table 2 LC-MS/MS analysis of methylation sites on ACE1$^{317\text{-}463aa}$ purified from *T. reesei*.

Supplementary figures

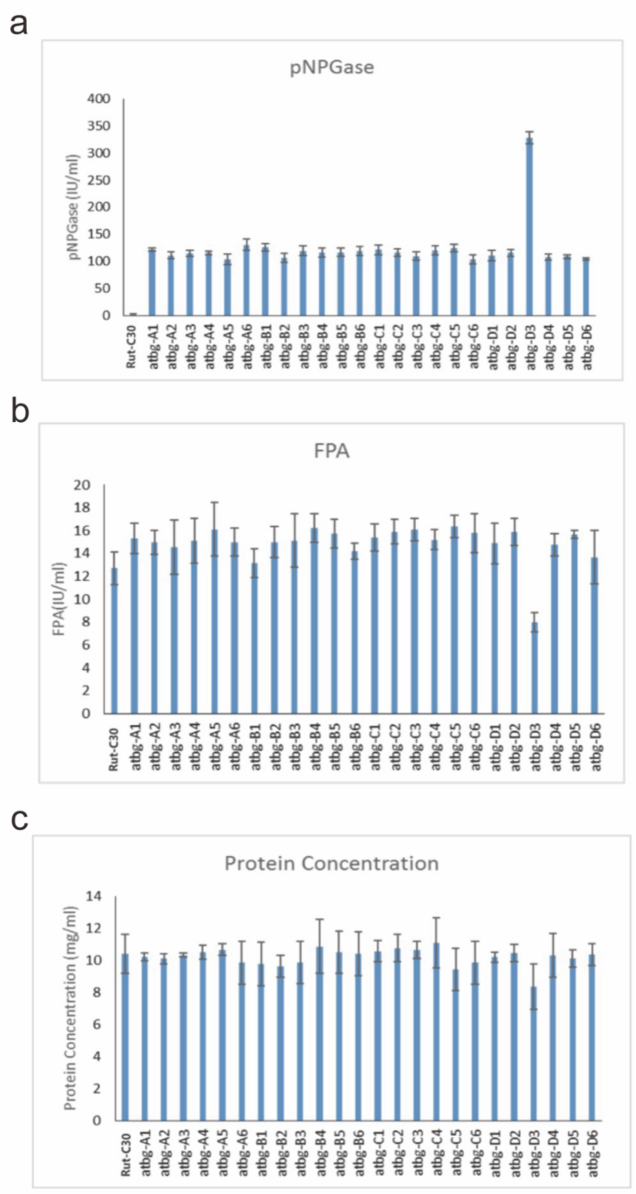

Supplementary Fig. 1: Evaluation of enzymatic activities and protein concentrations of the cellulase preparation of *T. reesei* Rut-C30 and its BglS transformants.

The β-glucosidase activities (a), FPAs (b) and protein concentrations (c) of culture supernatants from Rut-C30 and *bgls* random insertion transformants induced by 3% Avicel and 2% wheat bran. All the samples were tested by three replicates. Data are represented as mean ± SD.

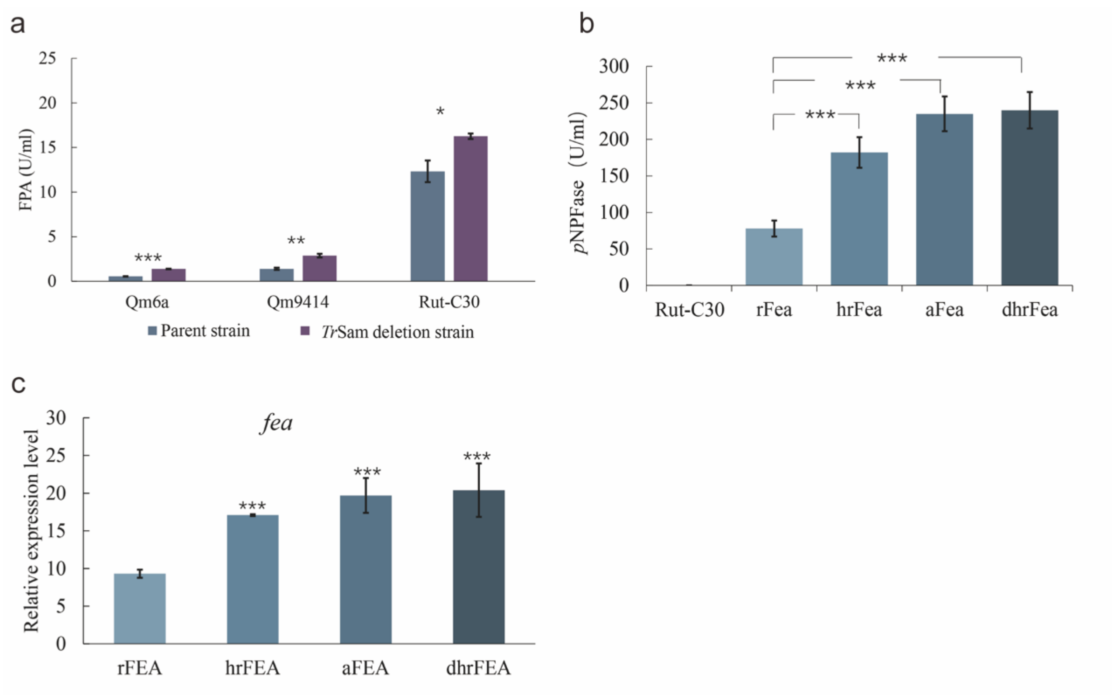

Supplementary Fig. 2: Evaluation of heterologous protein FEA production in *Tr*SAM deletion strain.

a FPAs of different *T. reesei* strains and their corresponding *trsam* deletion strains induced by 3% Avicel and 2% wheat bran.

b *p*NPFase activities of transformants with different the means of recombinant expressing of *fea*.

c Relative expression level of *fea* under inducing medium.

rFEA: random insertion of *fea*. hrFEA: homologous recombination in *cbh1* loci. aFEA: homologous recombination in the 48.8 kb fragment lost loci. dhrFEA: *trsam* deleted further in hrFEA. All the samples were tested by three replicates. Data are represented as mean ± SD * $P < 0.05$; ** $P < 0.01$; *** $P < 0.001$.

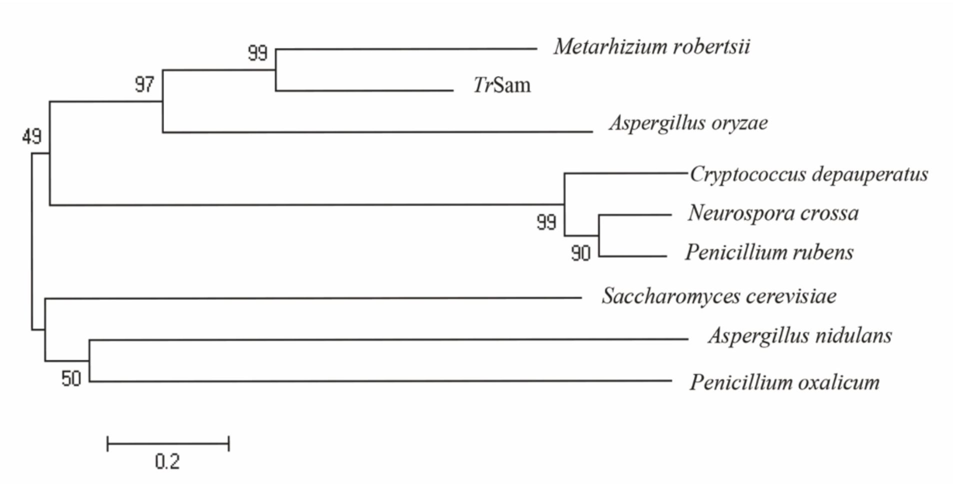

Supplementary Fig. 3: Phylogenetic tree of *Tr*SAM and its fungal orthologues.

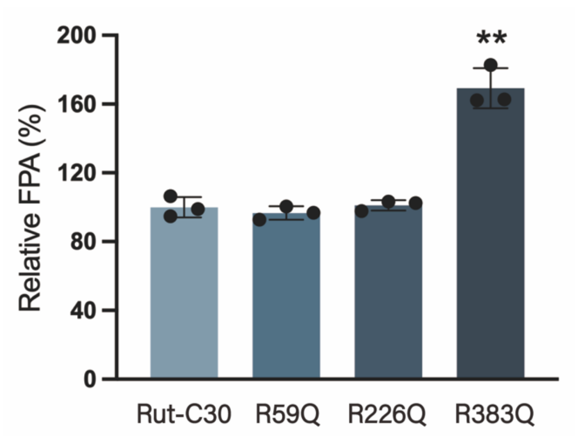

Supplementary Fig. 4: Evaluation of enzymatic activities of *T. reesei* and its transformants harboring ACE1 variants induced by 1% lactose. Rut-C30: parent strain. R59Q: strain harboring ACE1^{R59Q}. R226Q: strain harboring ACE1^{R226Q}. R383Q: strain harboring ACE1^{R383Q}. All the samples were tested by three replicates. Data are represented as mean ± SD. ** $P < 0.01$.

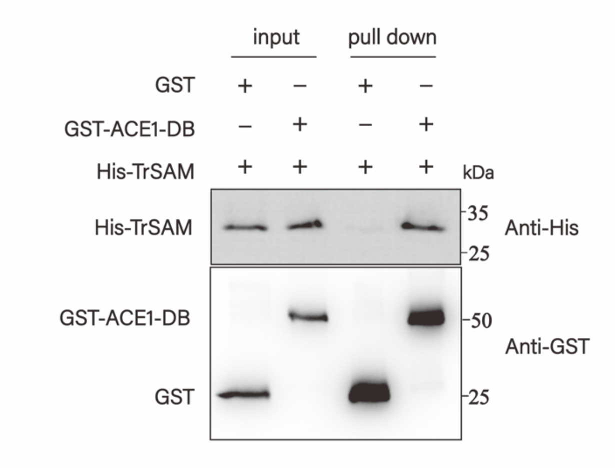

Supplementary Fig. 5: The interaction between ACE1 and *Tr*SAM was studied by *in vitro* pull-down assay. The molecular weights of GST-ACE-DB and His-TrSAM are 50 kDa and 33 kDa respectively.

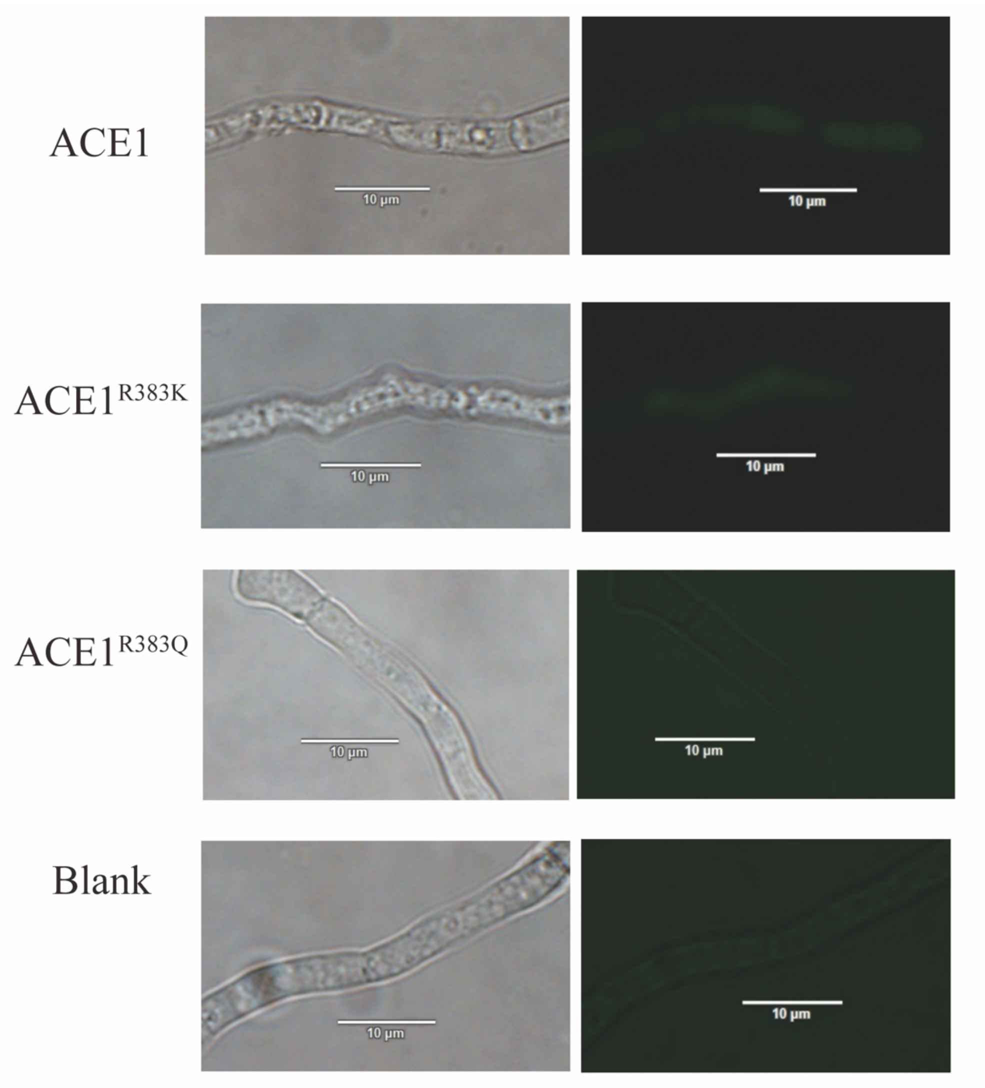

Supplementary Fig. 6: Analysis of interaction between *Tr*SAM and ACE1 variants induced by 1% lactose. ACE1: strain harboring wild type ACE1 fused with C-GFP and *Tr*SAM fused with N-GFP. $ACE1^{R383K}$: strain harboring $ACE1^{R383K}$ fused with C-GFP and *Tr*SAM fused with N-GFP. $ACE1^{R383Q}$: strain harboring $ACE1^{R383Q}$ fused with C-GFP and *Tr*SAM fused with N-GFP. Blank: wild type strain. All strains were cultured in MM medium containing 1% lactose as sole carbon source for 24 hours. Scale bars, 10 μm.

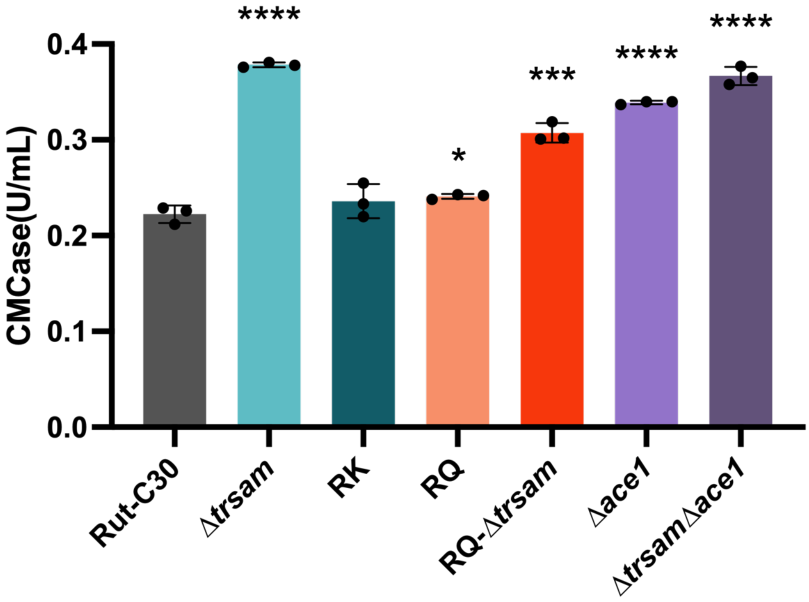

Supplementary Fig. 7: CMCase of *T. reesei* Rut-C30 and its mutants in repressing medium. Rut-C30: parent strain. Δ*trsam*: *trsam* deletion strain. RK: strain harboring ACE1^{R383K}. RQ: strain harboring ACE1^{R383Q}. RQ-Δ*trsam*: strain harboring ACE1^{R383Q} and deletion of *trsam*. Δ*ace1*: *ace1* deletion strain. Δ*trsam*Δ*ace1*: *trsam* and *ace1* double deletion strain. All strains were cultured in MM medium containing 1% glucose as sole carbon source for 24 hours. All the samples were tested by three replicates. Data are represented as mean ± SD. * $P < 0.05$; ** $P < 0.01$; *** $P < 0.001$; **** $P < 0.0001$.

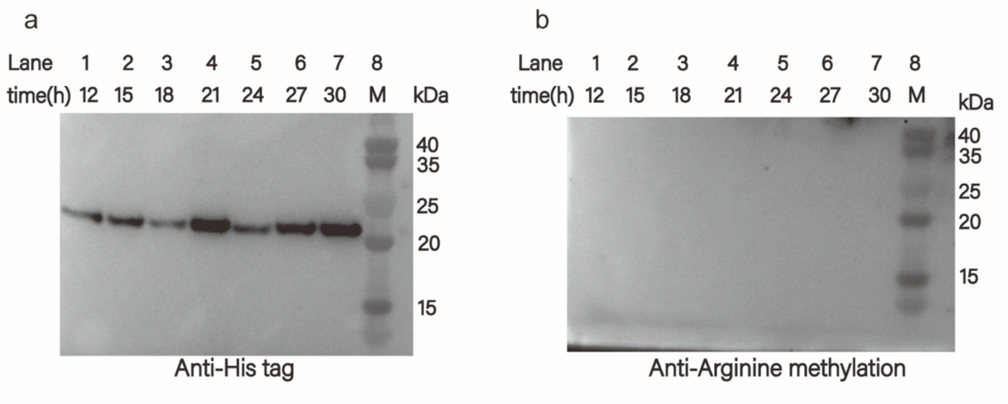

Supplementary Fig. 8: Detection of ACE1 methylation *in vivo*.

a Western blot analysis reflecting the expression of 6×His-ACE1$^{317\text{-}463aa}$. **b** Western blots analysis showing arginine methylation of 6×His-ACE1$^{317\text{-}463aa}$. Intracellular proteins were extracted from the transformants that overexpressed ACE1$^{317\text{-}463aa}$ after 12~30 h (3 h intervals) cultured with 2% glucose.

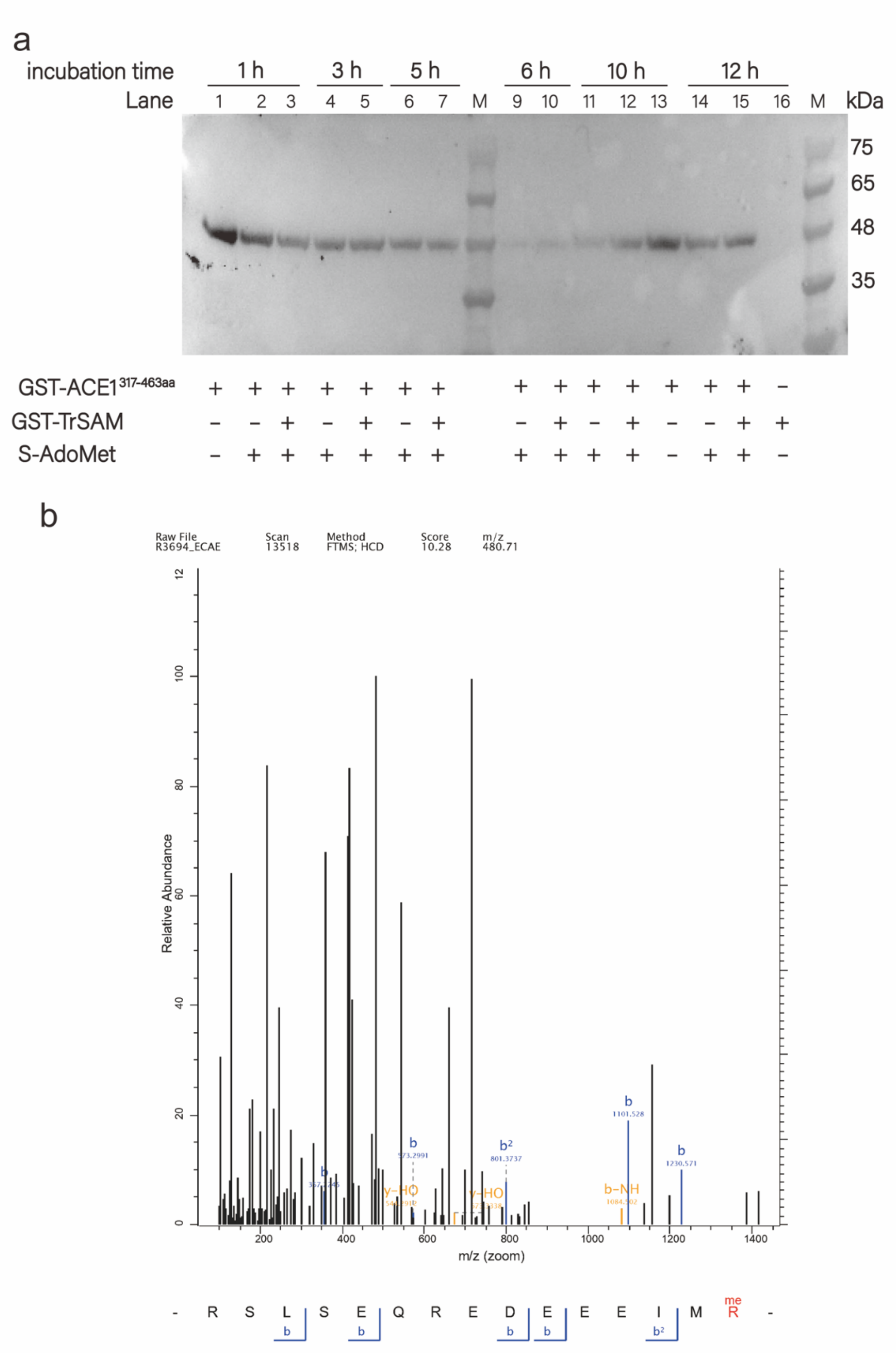

Supplementary Fig. 9: Detection of ACE1 methylation *in vitro*.

a Western blot analysis reflecting arginine methylation of recombinant GST-ACE1$^{317-463aa}$. The molecular weights of GST-ACE1$^{317-463aa}$ and GST-TrSAM are 46 kDa and 50 kDa respectively. S-AdoMet, S adenosine methionine. **b** LC-MS/MS analysis revealing the methylation of R383 of GST-ACE1$^{317-463aa}$ purified from *E.Coli* BL21(DE3).

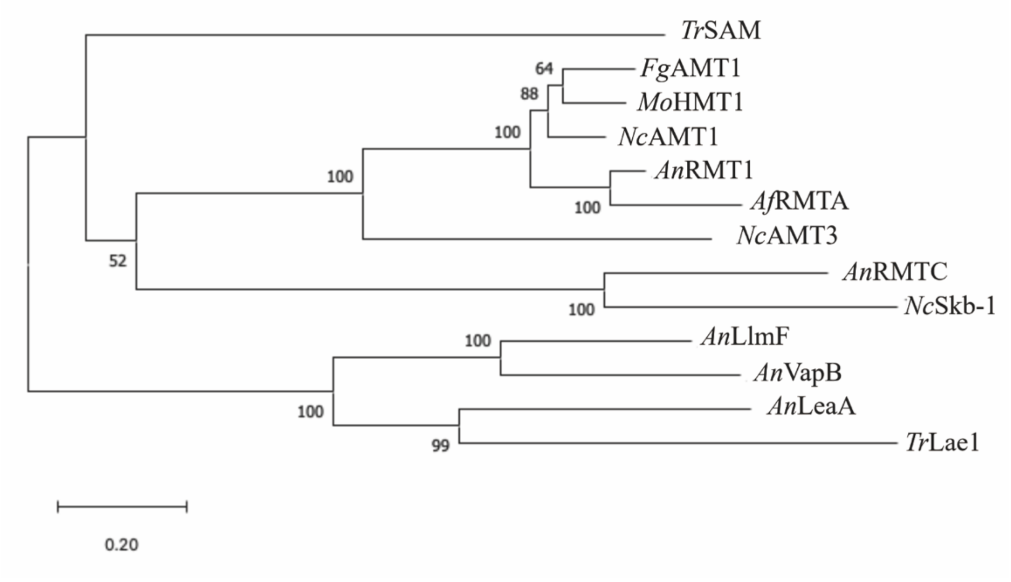

Supplementary Fig. 10: Phylogenetic tree of *Tr*SAM and well-investigated arginine methyltransferases in filamentous fungi. *Tr*: *Trichoderma reesei.* Fg: *Fusarium graminearum. Mo*: *Magnaporthe oryzae. Nc*: *Neurospora crassa. An*: *Aspergillus nidulans. Af*: *Aspergillus flavus.*

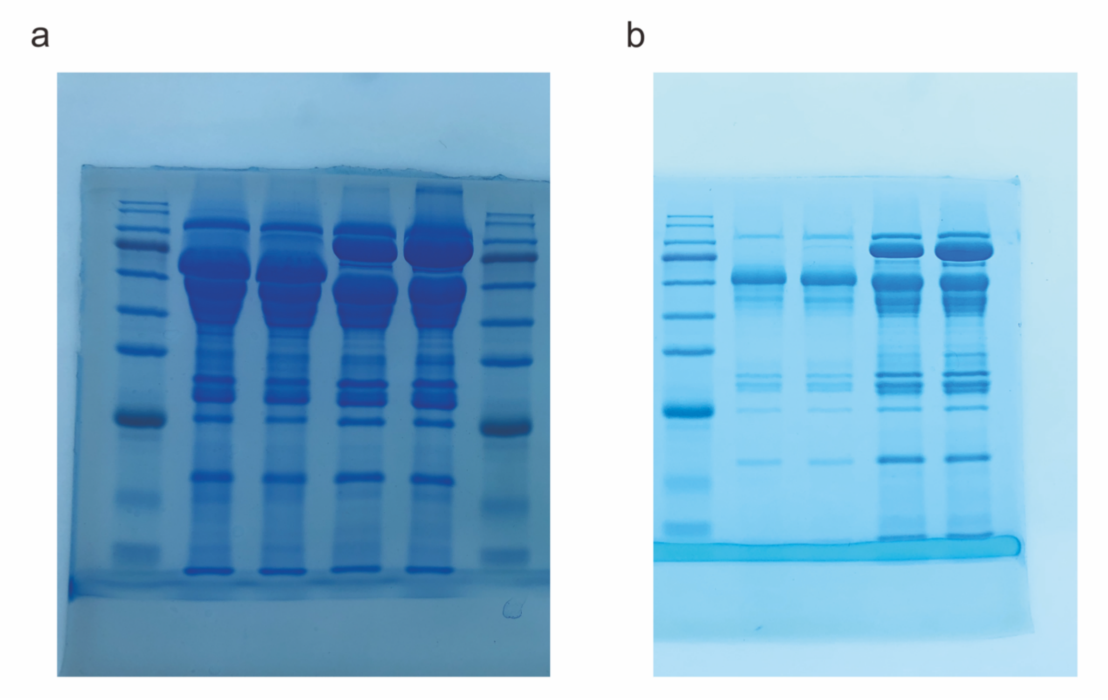

Supplementary Fig. 11: Source gel image of protein production by SDS-PAGE. **a** SDS-PAGE analysis of protein production under inducing condition. **b** SDS-PAGE analysis of protein production under repressing condition. Inducing condition: 3% Avicel and 2% wheat bran were used as carbon source. Repressing condition: inducing culture added with 2% Glucose. All of the samples were collected after 5-day fermentation. Lanes represent Rut-C30, atbg-A1, atbg-D3 and atbg-U10 from left to right.

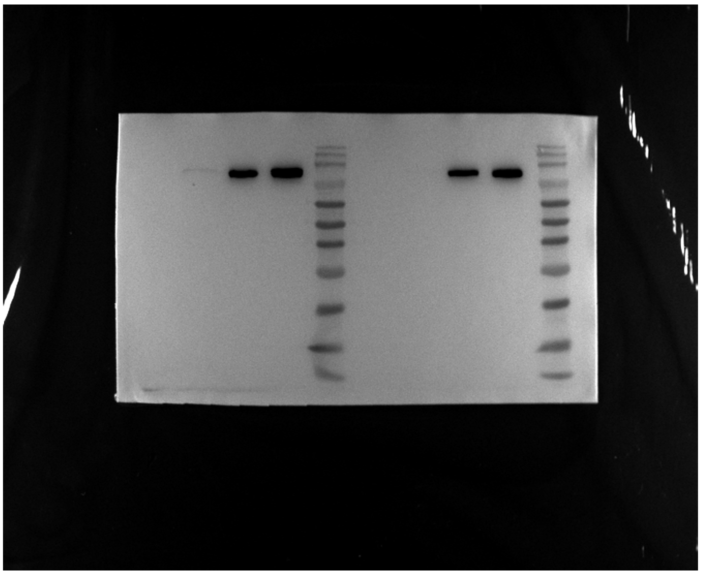

Supplementary Fig. 12: Source blot image of BglS production by Western blot.

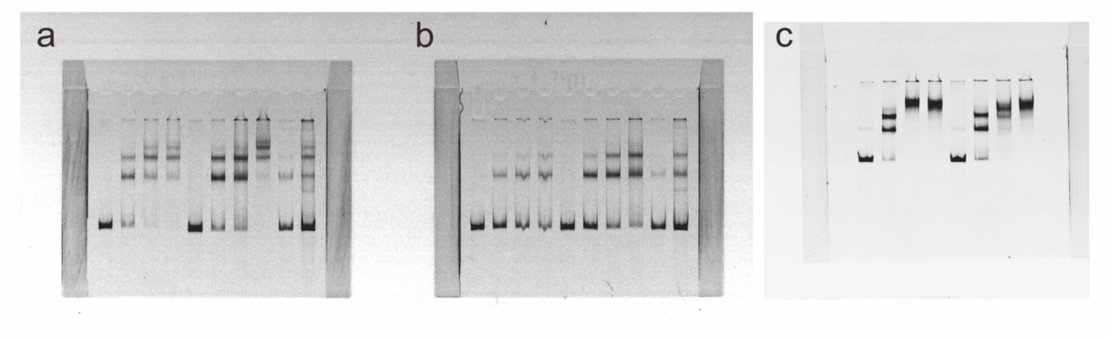

Supplementary Fig. 13: Source gel image of EMSA analysis.

a Comparative analysis of *cbh1* P3 binding ability of recombinant ACE1-DB.

b Comparative analysis of *cbh1* P3 binding ability of recombinant ACE1^{R383Q}-DB.

c Evaluation of the competitive *cbh1* P3 binding ability between recombinant ACE1-DB (or ACE1^{R383Q}-DB) and XYR1.

Supplementary tables

Supplementary Table 1 Prediction of putative methylation sites of ACE1

Position	Peptide	Met-Types[a]	Score
59	FVPPTLTRSQSAFDD	R. di[b]	4.23
226	LDVPRRIRSKEIICL	R. di	3.89
383	EDEEEIMRSMARRKK	R. di	4.66

[a] Predited on "http://msp.biocuckoo.org/"

[b] Di-methylation of arginine (symmetry or asymmetry)

Supplementary Table 2 LC-MS/MS analysis of methylation sites on ACE1$^{317\text{-}463aa}$ purified from *T. reesei.*

Position	Confidence of Dimethyl	Confidence of Methyl
R336	100	-
R342	-	100
K356	100	-
K368	96.3	-
K390	-	99.9
K400	100	-
K401	90.9	98.6
R413	-	100
K446	100	-
