# Supplementary material for: The protein methyltransferase TrSAM inhibits cellulase gene expression by interacting with the negative regulator ACE1 in Trichoderma reesei
Source: Commun Biol. 2024 Mar 28;7:375. doi: 10.1038/s42003-024-06072-1 (PMC10978942; doi:10.1038/s42003-024-06072-1)
Supplement: Supplementary file 2 — Description of Additional Supplementary Files [file 42003_2024_6072_MOESM2_ESM.pdf]

Description of Additional Supplementary Files

File name: Supplementary Data 1
Description: Oligonucleotides used in this study.

File name: Supplementary Data 2
Description: The source data underlying the graphs in the paper.
