# Supplementary material for: The protein methyltransferase TrSAM inhibits cellulase gene expression by interacting with the negative regulator ACE1 in Trichoderma reesei
Source: Commun Biol. 2024 Mar 28;7:375. doi: 10.1038/s42003-024-06072-1 (PMC10978942; doi:10.1038/s42003-024-06072-1)
Supplement: Supplementary file 3 — Supplementary Data 1 [file 42003_2024_6072_MOESM3_ESM.pdf]

Supplementary Data 1. Oligonucleotides used in this study

Oligonucleotides	Sequence (5'- 3')	Notes
gRNA1	GGCCGTCATCTCGGCCTTCT	Spacer sequences of gRNA for gene knock out by CRISPR/Cas9
gRNA2	GGTCTCCAACAGTGCTTTCC	
gRNA3	GGAGGCAGTAACCTTCTTGC	
gRNA4	GGCAGTTGGGTAATCCCACA	
gRNA5	GGTATTGCTTGTCAGCAATT	
gRNA6	GGTGTACGCGCCGAAGACGT	
gRNA7	GGCATATTATGGGCAACCAT	
gRNA8	GCTCTGGAATTGGTACACGG	
gRNA9	GGCATCCAGACGTGTGCAAT	
gRNA10	GGACCTACAAAGGCCTGACG	
gRNA11	GGGATCTTCACCATTGCTCA	
gRNA12	GGCTTTCTGGTATGGCCAGT	
gRNA13	GGTCCACAAGCTTCGCAAGA	
gRNA14	GGAAAGGCACAATTCGTGTT	
gRNA15	GGCATTTGCGAGATTCATGG	
gRNA16	GGTCTTCATGCGCAATACTG	
gRNA17	GGTATATACGCGCATACTAT	
1-5F	TACCCGGGGATCCTCTAGAGATTAATCAAGTGGCTAAACGTAC	Primers for donor DNA for *cbh1* deletion
1-5R	GTATACTGGCTTAACTATGCGGCAACTTCCGATACATGATGCGC	
1-loopF	GAAAATTCCGTCACCAGCCCTGCTATGAGAAATTCTGGAGACGGC	
1-loopR	GTACAATTATTAGCCGCTGG	
1-3F	CCAGCGGCTAATAATTGTACCATCCCGTTCATAACCCGTAGAATC	
1-3R	GCATGCCTGCAGGTCGACGATGCCAGAGTATCTGTTGGACGC	
2-5F	TACCCGGGGATCCTCTAGAGATTCGACACCTAGCTATCGCTACC	Primers for donor DNA for an unknown protein (2) coding gene deletion
2-5R	GTATACTGGCTTAACTATGCGGCCTTGTCCGTTGCGAGGCCAAC	
2-loopF	GAAAATTCCGTCACCAGCCCTGGCGGCTGAATCCATTCAGTCAG	
2-loopR	TGCACGCACATAGGCCGAAATGC	
2-3F	GCATTTCGGCCTATGTGCGTGCAGGCCGCCAGCTATGGTCTCAC	
2-3R	GCATGCCTGCAGGTCGACGATCTGAACCCTTACTACTCTCAG	
3-5F	TACCCGGGGATCCTCTAGAGATTGCCGCATTACTAGCAGACTATTC	Primers for donor DNA for *pfs* (3) coding gene deletion
3-5R	GTATACTGGCTTAACTATGCGGCACATCACCCCGGGCAGCGG	
3-loopF	GAAAATTCCGTCACCAGCCCTGGAATTGGTCAAAGACAGAGTCTTGGC	
3-loopR	CAGTGCAAGAGGACACTGGATGCTC	
3-3F	GAGCATCCAGTGTCCTCTTGCACTGCAAATACAGTAACAAATGGGGACCAG	
3-3R	GCATGCCTGCAGGTCGACGATGCCATCAGGTGCCAAGTGGTTG	
4-5F	TACCCGGGGATCCTCTAGAGATTCCCTTCCCGAACTGTTAGACGGG	Primers for donor DNA for *swo1* (4) deletion
4-5R	GTATACTGGCTTAACTATGCGGCGCGTTCTTGTGGGCGGCTAC	
4-loopF	GAAAATTCCGTCACCAGCCCTGTTAATAGATACGTACCCTTGCAATTGG	
4-loopR	CAACTGACAGCTCTACTATAAGCGAAC	
4-3F	GTTCGCTTATAGTAGAGCTGTCAGTTGGGGATCAGGGCCCTTTAACTTGCCTG	

4-3R	GCATGCCTGCAGGTCGACGATCCACAGGGCTGGGTGGGAGTC	
5-5F	TACCCGGGGATCCTCTAGAGATTGGATCCATCAATGCCACCG	Primers for donor DNA for *xyn4* (5) deletion
5-5R	GTATACTGGCTTAACTATGCGGCCTCATTTCAAAGTGAGCATGCTG	
5-loopF	GAAAATTCCGTCACCAGCCCTGACTCAACAATTGATACACAGGCAC	
5-loopR	CAGGATTGTATGACTCGCTTCA	
5-3F	TGAAGCGAGTCATACAATCCTGCATGTCGTCAATTCTGCGG	
5-3R	GCATGCCTGCAGGTCGACGATTTAGTTAACTTGCCGTGACAACC	
6-5F	TACCCGGGGATCCTCTAGAGATTGCTCAGCCCAGAGGGTCCATC	Primers for donor DNA for an unnamed protein (6) coding gene deletion
6-5R	GTATACTGGCTTAACTATGCGGCACTTGTGCCATCTCCCAATC	
6-loopF	GAAAATTCCGTCACCAGCCCTGCGATCCGATCCAGGAGAGCGAGG	
6-loopR	AGGCTGGTCCTCTTTCTTGTGAAGAATG	
6-3F	CATTCTTCACAAGAAAGAGGACCAGCCTGGGACAATTCATTGCCGAAC	
6-3R	GCATGCCTGCAGGTCGACGATCGAGCGACGTGAAATGGATTGAG	
7-5F	TACCCGGGGATCCTCTAGAGATTTTGTCGGTGCCACTGTCGAA	Primers for donor DNA for ferric reductase (7) coding gene deletion
7-5R	GTATACTGGCTTAACTATGCGGCCCACCCTTATCAGGCACATTTGA	
7-loopF	GAAAATTCCGTCACCAGCCCTGAGTGAATCTAGGGTACGAGAC	
7-loopR	CTGGTGGAAGAACTCAAGGATC	
7-3F	GATCCTTGAGTTCTTCCACCAGTGAAACAGGTTTATCCCCAGG	
7-3R	GCATGCCTGCAGGTCGACGATCCGTTGAAAGCGTGAAAGCTG	
8-5F	TACCCGGGGATCCTCTAGAGATTGCATGACGAGCAGCCAGATAAG	Primers for donor DNA for copper transporter (8) coding gene deletion
8-5R	GTATACTGGCTTAACTATGCGGCCTAGGGTCACTGTTAGCAAAC	
8-loopF	GAAAATTCCGTCACCAGCCCTGCTAGAACCACAACTTGTAAGCG	
8-loopR	TTATATTAAGTTCTCTGTGCTG	
8-3F	CAGCACAGAGAACTTAATATAAGCGTCTGGGTCATTCCCTGAAAG	
8-3R	GCATGCCTGCAGGTCGACGATGGTGCGGGAATGATGTGTGGTG	
9-5F	TACCCGGGGATCCTCTAGAGATTTGGTTGCATCGGTCGAGCT	Primers for donor DNA for lipase (9) coding gene deletion
9-5R	GTATACTGGCTTAACTATGCGGCTATACTCAAGACACTACCGGG	
9-loopF	GAAAATTCCGTCACCAGCCCTGGCTTGATGCGGATTCTTGAAGG	
9-loopR	GTAGGACTATTCCAATGCACG	
9-3F	CGTGCATTGGAATAGTCCTACCCTACTTCTTACCCTGTCC	
9-3R	GCATGCCTGCAGGTCGACGATTGCAATACCATGGACGACG	
10-5F	TACCCGGGGATCCTCTAGAGATTCAATGGGAGATCGAGAGCCG	Primers for donor DNA for lipase (10) coding gene deletion
10-5R	GTATACTGGCTTAACTATGCGGCAGCAATTCTCCTTGTCGTCGT	
10-loopF	GAAAATTCCGTCACCAGCCCTGGATACCTGCTGGAGTCTATAAG	
10-loopR	AACCCATCGTCGCTTCCACC	
10-3F	GGTGGAAGCGACGATGGGTTGAGAAACTCAAAGCAGTCATTG	
10-3R	GCATGCCTGCAGGTCGACGATGCATCAAGAGCCTCTGCATCCTTG	
11-5F	TACCCGGGGATCCTCTAGAGATTGAATAAGGTAGCGCCTGTACG	Primers for donor DNA for mannitol 1-phosphate dehydrogenase
11-5R	GTATACTGGCTTAACTATGCGGCGCGAGTACTAGTTGTACAGACG	
11-loopF	GAAAATTCCGTCACCAGCCCTGAAGTCCCATGGCAGCCTCAAAT	
11-loopR	CACAGGCAGCTCAAGATGCAAC	
11-3F	GTTGCATCTTGAGCTGCCTGTGGCCATCCATTGTCATCAGTTG	

11-3R	GCATGCCTGCAGGTCGACGATCCTTATAGACTCCAGCAGG	(11) coding gene deletion
12-5F	TACCCGGGGATCCTCTAGAGATTCCATGACGGCAACTGCCATC	Primers for donor DNA for MFS transporter (12) coding gene deletion
12-5R	GTATACTGGCTTAACTATGCGGCTTGATGTTGATACTACAAGTGCG	
12-loopF	GAAAATTCCGTCACCAGCCCTGCTGAGACCAACGGCATCTGACTG	
12-loopR	GCTTGTTGTGATGGACTTATGCAAG	
12-3F	CTTGCATAAGTCCATCACAACAAGCGGAACACTCTTATGGACGTTG	
12-3R	GCATGCCTGCAGGTCGACGATGGCCGGTTATGTACACTCATTC	
13-5F	TACCCGGGGATCCTCTAGAGATTCTTCAACAACACCATTTGGTTAG	Primers for donor DNA for integral membrane protein (13) coding gene deletion
13-5R	GTATACTGGCTTAACTATGCGGCATACCTACTTCGTACGAGATC	
13-loopF	GAAAATTCCGTCACCAGCCCTGAAGAGGCCGTAGCATATACC	
13-loopR	GCGTGAATATTTAGCCTCGAC	
13-3F	GTCGAGGCTAAATATTCACGCTTGCTAAAGCCTGCGCACA	
13-3R	GCATGCCTGCAGGTCGACGATTTACTGACTTCCTAGCGGC	
14-5F	TACCCGGGGATCCTCTAGAGATTTGGCTGTTCCGACGTTTACTG	Primers for donor DNA for p450 (14) coding gene deletion
14-5R	GTATACTGGCTTAACTATGCGGCTATACAAGCCCCACGGTGATC	
14-loopF	GAAAATTCCGTCACCAGCCCTGCTGATCAAGTCGCTGAACG	
14-loopR	ACTGTGCCTCCAGGGATGAA	
14-3F	AATTCATCCCTGGAGGCACAGTATACGCGCTCAAACTGTAACTG	
14-3R	GCATGCCTGCAGGTCGACGATAGTGTCTTGCTGAGTTCTTTCG	
15-5F	TACCCGGGGATCCTCTAGAGATTCTTGTGAAGTCCACTCAAACGCC	Primers for donor DNA for *trsam* (15) deletion
15-5R	GTATACTGGCTTAACTATGCGGCGGATTCGTATGAGGCATGTGC	
15-loopF	GAAAATTCCGTCACCAGCCCTGCTCACTTGGGAATATTGGTCG	
15-loopR	CGAAAGCTTTGAGATGCCAATG	
15-3F	CATTGGCATCTCAAAGCTTTCGGTAACGGTAGAACGCTTGAGG	
15-3R	GCATGCCTGCAGGTCGACGATCCTTTCCTGCACACGCCTACGG	
16-5F	TACCCGGGGATCCTCTAGAGATTGAAACGCTGTCGGCTTCAAGGCAG	Primers for donor DNA for an unnamed protein (16) coding gene deletion
16-5R	GTATACTGGCTTAACTATGCGGCGCCAGCTGTTCAACACCGTTC	
16-loopF	GAAAATTCCGTCACCAGCCCTGAAGTATACTACGTACGTGGGAC	
16-loopR	GTATCGCCGAGGATGAATATTCACTG	
16-3F	CAGTGAATATTCATCCTCGGCGATACTACGGGAAGCAAGGCGTTGAAG	
16-3R	GCATGCCTGCAGGTCGACGATGTACTATTTAGCGACAACTTGTG	
17-5F	TACCCGGGGATCCTCTAGAGATTGGTAGTCTTCGATCACAACCTC	Primers for donor DNA for a hypothetical GPCR (17) coding gene deletion
17-5R	GTATACTGGCTTAACTATGCGGCCTCCCACTCCCTCAGATAGAC	
17-loopF	GAAAATTCCGTCACCAGCCCTGGCTTCCGATGCTGCTACTTTC	
17-loopR	GGCGCGAAAGAAAGCTGATCC	
17-3F	GGATCAGCTTTCTTTCGCGCCGTCTGGCAAATTTCCTGGCCCG	
17-3R	GCATGCCTGCAGGTCGACGATACTATCCTGCTCAACCTGGCAAG	
Cy5-labeled	ACTAACTCGCGTACTG	Primers for electrophoretic mobility shift assay
Cy5-cbh1P1F	ACTAACTCGCGTACTGTTAACCCAGACTGACCGGA	
Cy5-cbh1P1R	ACTAACTCGCGTACTGACAATTATTAGCCGCTGGTA	
Cy5-cbh1P2F	ACTAACTCGCGTACTGACAATCAAGTGGCTAAACGT	

Cy5-cbh1P2R	ACTAACTCGCGTACTGCATTTCAATATGGACCACTT	
Cy5-cbh1P3F	ACTAACTCGCGTACTGATGTAAGTCGGCACTGAACA	
Cy5-cbh1P3R	ACTAACTCGCGTACTGATGAGTAGATGGGGAGAGCA	
Cy5xyr1P1F	ACTAACTCGCGTACTGGCCGTCCAATTACATGCCTC	
Cy5xyr1P1R	ACTAACTCGCGTACTGACTGCATCTGTGCGAGGCA	
Cy5xyr1P2F	ACTAACTCGCGTACTGGTTCTTACTACGGCAATAAA	
Cy5xyr1P2R	ACTAACTCGCGTACTGGAGGGGGGGAGGCAAGTC	
Cy5-act1F	ACTAACTCGCGTACTGACGGACAACGATGTCATCAT	
Cy5-act1R	ACTAACTCGCGTACTGCCGAGCTCAGACCTGTACAAT	
Cy5-xyr1P6F	ACTAACTCGCGTACTGGCTTCATACCCGCCTGCACTC	
Cy5-xyr1P6R	ACTAACTCGCGTACTGCGGCTGCCACATCACGATCG	
4TACE1F	ATCTGGTTCCGCGTGGATCCATGGACATTGACCCATCTG	Primers for ACE1,XYR1,CRZ1 DNA-binding domain and SAM-GST expression and purification
4TACE1R	CCCGGGAATTCCGGGGATCCGTACATGGCCGGGGCGTCC	
4TSAMF	ATCTGGTTCCGCGTGGATCCATGTCCCAATACGACTCCAT	
4TSAMR	TCGAGTCGACCCGGGAATTCCGGGGATCCTTACAACC	
	GCAACATGATAACCAGTTCGTTGCGTGGGAAATACTGCCGTAG	
4TXYR1F	CGGGATCCGAGCTTTCGAGTTCACGCATG	
4TXYR1R	TTTCTCGAGCTCAATGTGGCCATGAG	
4TCRZ1F	ATCTGGTTCCGCGTGGATCCGCGACCTTTCAGTGTACCT	
4TCRZ1R	CGAGTCGACCCGGGAATTCCTGCCACTGGCGTTGCCTTT	
ΔSAMupF	atgcctgcaggtcgacgattTGTTTCATTCGCCGATCCAG	Primers for *trsam* gene deletion and verification
ΔSAMupR	tactggcttaactatgcggcCATGAACTATTTGTTTGCTT	
ΔSAMUF	AAGCAAACAAATAGTTCATGgccgcatagttaagccagta	
ΔSAMUR	GAGCGTCTTCCTGGATCGCCcagggctggtgacggaattt	
ΔSAMloopF	aaattccgtcaccagccctgGGCGATCCAGGAAGACGCTC	
ΔSAMloopR	AGCTCAGCTCTTCTACATACTTGTACTGAAGCGCTATGTG	
ΔSAMdwF	CACATAGCGCTTCAGTACAAGTATGTAGAAGAGCTGAGCT	
ΔSAMdwR	cccggggatcctctagagatCAAGTGACCGCGTTTCGATT	
Sam-VF	GAGCATCCGGCTATCAAAGG	
Sam-VR	CCTGTAGGGTCTGAAGTATT	
OEsamPF	cgacggccagtgccaagcttAGGACTTCCAGGGCTACTTG	Primers for *trsam* gene overexpression and complementation
OEsamPR	ATGGAGTCGTATTGGGACATGATTGTGCTGTAGCTGCGCT	
OEsamF	AGCGCAGCTACAGCACAATCATGTCCCAATACGACTCCAT	
OEsamR	ATGGAGTCGTATTGGGACATGATTGTGCTGTAGCTGCGCT	
OEsamTF	AAGAGAACAAATCGTCACCATCACCATCACCATTAACCCGGCATGAAGT	
OEsamTR	ATGGAGTCGTATTGGGACATGATTGTGCTGTAGCTGCGCT	
resamPF	cgacggccagtgccaagcttCGAGAGCATGCTGGCTATTG	
resamPR	TGGAGTCGTATTGGGACATCATGAACTATTTGTTTGCTT	
resamF	AAGCAAACAAATAGTTCATGATGTCCCAATACGACTCCA	
resamR	CCTGTTACTACTCTCAGCTTAATGGTGATGGTGATGGTGACGATTTGTTCTCT	
resamTF	AGAGAACAAATCGTCACCATCACCATCACCATTAAGCTGAGAGTAGTAACAGG	
resamTR	CCTGTTACTACTCTCAGCTTAATGGTGATGGTGATGGTGACGATTTGTTCTCT	

ΔACE1upF	gcctgcaggtcgacgattTCAGCCCACCCCCGCGCTATG	Primers for *trsam* gene deletion and verification
ΔACE1upR	tactggcttaactatgcggcGGCGGCCGAGATCTGTGTT	
ΔACE1UF	AACACAGATCTCGGCCGCCgccgcatagttaagccagta	
ΔACE1UR	CCTACCTATTACCAGGTACcagggctggtgacggaattt	
ΔACE1loopF	aaattccgtcaccagccctgGTACCTGGTAATAGGTAGG	
ΔACE1loopR	ATTGTATGTGTCGCAATCTTTGGCGTAAGCGCTGCGGAG	
ΔACE1dwF	CTCCGCAGCGCTTACGCCAAAGATTGCGACACATACAAT	
ΔACE1dwR	acccggggatcctctagagatATGAAAGATTCTTCTCTT	
Ace1-VF	GGCGTCGCCAGCGCGTGTGC	
Ace1-VR	GTTGATCAATTACACAGAGG	
RT-cbh1F	GCGGATCCTCTTTCTCAG	Primers for quantitative RT-PCR
RT-cbh1R	ATGTTGGCGTAGTAATCATCC	
RT-xyr1F	GAGTATCAGCGCAACTTTAGCA	
RT-xyr1R	CATCGGTATAGTGCAAGAAGCTC	
AD-SAMF	GCCATGGAGGCCAGTGAATTCATGTCCCAATACGACTCCA	Primers for yeast two-hybrid
AD-SAMR	ATGCCCACCCGGGTGGAATTCCTACAACCGCAACATGATA	
BD-SAMF	ATGGCCATGGAGGCCGAATTCATGTCCCAATACGACTCCA	
BD-SAMR	CGACGGATCCCCGGGAATTCCTACAACCGCAACATGATAA	
AD-ACE1F	GCCATGGAGGCCAGTGAATTCATGGACATTGACCCATCTG	
AD-ACE1R	ATGCCCACCCGGGTGGAATTCTTAGTACATGGCCGGGGCG	
BD-ACE1F	ATGGCCATGGAGGCCGAATTCATGGACATTGACCCATCTG	
BD-ACE1R	CGACGGATCCCCGGGAATTCTTAGTACATGGCCGGGGCGT	
AD-Xyr1F	GCCATGGAGGCCAGTGAATTCATGTTGTCCAATCCTCTCC	
AD-Xyr1R	ATGCCCACCCGGGTGGAATTCTTAATGATGATGATGATGATGGAGGGCCAGAC	
BD-Xyr1F	ATGGCCATGGAGGCCGAATTCATGTTGTCCAATCCTCTCC	
BD-Xyr1R	TCGACGGATCCCCGGGAATTCTTAATGATGATGATGATGATGGAGGGCCAGAC	
