# Supplementary material for: The protein methyltransferase TrSAM inhibits cellulase gene expression by interacting with the negative regulator ACE1 in Trichoderma reesei
Source: Commun Biol. 2024 Mar 28;7:375. doi: 10.1038/s42003-024-06072-1 (PMC10978942; doi:10.1038/s42003-024-06072-1)
Supplement: Supplementary file 5 — Reporting Summary [file 42003_2024_6072_MOESM5_ESM.pdf]

nature portfolio

Corresponding author(s): Zhihua Zhou

Last updated by author(s): Jan 25, 2024

Reporting Summary

Nature Portfolio wishes to improve the reproducibility of the work that we publish. This form provides structure for consistency and transparency in reporting. For further information on Nature Portfolio policies, see our Editorial Policies and the Editorial Policy Checklist.

Statistics

For all statistical analyses, confirm that the following items are present in the figure legend, table legend, main text, or Methods section.

n/a	Confirmed	
☐	☒	The exact sample size (*n*) for each experimental group/condition, given as a discrete number and unit of measurement
☐	☒	A statement on whether measurements were taken from distinct samples or whether the same sample was measured repeatedly
☒	☐	The statistical test(s) used AND whether they are one- or two-sided<br>*Only common tests should be described solely by name; describe more complex techniques in the Methods section.*
☒	☐	A description of all covariates tested
☒	☐	A description of any assumptions or corrections, such as tests of normality and adjustment for multiple comparisons
☐	☒	A full description of the statistical parameters including central tendency (e.g. means) or other basic estimates (e.g. regression coefficient) AND variation (e.g. standard deviation) or associated estimates of uncertainty (e.g. confidence intervals)
☒	☐	For null hypothesis testing, the test statistic (e.g. *F*, *t*, *r*) with confidence intervals, effect sizes, degrees of freedom and *P* value noted<br>*Give P values as exact values whenever suitable.*
☒	☐	For Bayesian analysis, information on the choice of priors and Markov chain Monte Carlo settings
☒	☐	For hierarchical and complex designs, identification of the appropriate level for tests and full reporting of outcomes
☒	☐	Estimates of effect sizes (e.g. Cohen's *d*, Pearson's *r*), indicating how they were calculated

Our web collection on statistics for biologists contains articles on many of the points above.

Software and code

Policy information about availability of computer code

Data collection	Enzyme activities data were collected from Thermo Scientific Microplate Reader Varioskan Flush using SkanIt software. Bimolecular fluorescence complementation data were collected from fluorescence microscope Olympus BX53. Electrophoretic mobility shift assay data were collected from Image scanning analyzer Fuji Film FLA-9000.
Data analysis	The phylogenetic tree was analyzed with MEGA-X software. Image J software was used to analyze the gray values of protein bands revealed by immunoblotting.

For manuscripts utilizing custom algorithms or software that are central to the research but not yet described in published literature, software must be made available to editors and reviewers. We strongly encourage code deposition in a community repository (e.g. GitHub). See the Nature Portfolio guidelines for submitting code & software for further information.

Data

Policy information about availability of data

All manuscripts must include a data availability statement. This statement should provide the following information, where applicable:

- Accession codes, unique identifiers, or web links for publicly available datasets
- A description of any restrictions on data availability
- For clinical datasets or third party data, please ensure that the statement adheres to our policy

All data supporting the findings of this study are available within the paper and its Supplementary Information. Primer sequences used in this study are provided in Supplementary Table 3.

Human research participants

Policy information about studies involving human research participants and Sex and Gender in Research.

Reporting on sex and gender	not applicable
Population characteristics	not applicable
Recruitment	not applicable
Ethics oversight	not applicable

Note that full information on the approval of the study protocol must also be provided in the manuscript.

Field-specific reporting

Please select the one below that is the best fit for your research. If you are not sure, read the appropriate sections before making your selection.

☒ Life sciences ☐ Behavioural & social sciences ☐ Ecological, evolutionary & environmental sciences

For a reference copy of the document with all sections, see nature.com/documents/nr-reporting-summary-flat.pdf

Life sciences study design

All studies must disclose on these points even when the disclosure is negative.

Sample size	Biological triplicates were chosen as the standard for all experiments.
Data exclusions	No data exclusion.
Replication	All determinations were repeated in biological triplicates.
Randomization	The transformants and mutant strains were selected randomly for the analysis of phenotypic test.
Blinding	Group allocation is not involved in this study.

Reporting for specific materials, systems and methods

We require information from authors about some types of materials, experimental systems and methods used in many studies. Here, indicate whether each material, system or method listed is relevant to your study. If you are not sure if a list item applies to your research, read the appropriate section before selecting a response.

Materials & experimental systems

n/a	Involved in the study
☐	☒ Antibodies
☒	☐ Eukaryotic cell lines
☒	☐ Palaeontology and archaeology
☒	☐ Animals and other organisms
☒	☐ Clinical data
☒	☐ Dual use research of concern

Methods

n/a	Involved in the study
☒	☐ ChIP-seq
☒	☐ Flow cytometry
☒	☐ MRI-based neuroimaging

Antibodies

Antibodies used

Commercial antibodies: anti-dimethyl arginine mouse monoclonal antibody (1/500 dilution, Abcam, #ab413); anti-His tag mouse monoclonal anibody (1/5000 dilution, Yeasen, Shanghai, China, #30401ES) ; Peroxidase AffiniPure Goat Anti-Mouse antibody (1/5000 dilution, Yeasen, Shanghai, China, #33201ES); anti-GST mouse monoclonal antibody (1/5000 dilution, Yeasen, Shanghai, China, #30901ES).

Validation

For anti-dimethyl arginine mouse monoclonal antibody, validation with Westernblot is reported on the product page (https://www.abcam.cn/products/primary-antibodies/dimethyl-arginine-antibody-21c7-ab413.html?productWallTab=Abreviews); For ptotein tag antibodies, validation was performed using a combination of target protein. All data is reported with in the manuscript and supplemental materials.
